# An Alternating Current Electroosmotic Flow‐Based Ultrasensitive Electrochemiluminescence Microfluidic System for Ultrafast Monitoring, Detection of Proteins/miRNAs in Unprocessed Samples

**DOI:** 10.1002/advs.202307840

**Published:** 2023-12-09

**Authors:** Huiwen Xiong, Chenxin Zhu, Changhao Dai, Xin Ye, Yuanyuan Li, Pintao Li, Shuang Yang, Ghazala Ashraf, Dacheng Wei, Hui Chen, Huali Shen, Jilie Kong, Xueen Fang

**Affiliations:** ^1^ Department of Chemistry Fudan University Shanghai 200438 P. R. China; ^2^ Institutes of Biomedical Sciences and Minhang Hospital Fudan University Shanghai 200032 P. R. China; ^3^ State Key Laboratory of Molecular Engineering of Polymers Department of Macromolecular Science Fudan University Shanghai 200438 P. R. China; ^4^ Department of Laboratory Medicine The First Affiliated Hospital of Xi'an Jiaotong University Xi'an Shaanxi 710061 P. R. China; ^5^ Yizheng Hospital of Traditional Chinese Medicine Yangzhou 211400 P. R. China

**Keywords:** AC electroosmotic flow, acute diseases, biotic fluids, electrochemiluminescence‐microfluidic system, point‐of‐care testing

## Abstract

Early diagnosis of acute diseases is restricted by the sensitivity and complex process of sample treatment. Here, an ultrasensitive, rapid, and portable electrochemiluminescence‐microfluidic (ECL‐M) system is described via sandwich‐type immunoassay and surface plasmonic resonance (SPR) assay. Using a sandwich immunoreaction approach, the ECL‐M system employs cardiac troponin‐I antigen (cTnI) as a detection model with a Ru@SiO_2_ NPs labeled antibody as the signal probe. For miR‐499‐5p detection, gold nanoparticles generate SPR effects to enhance Ru(bpy)_3_^2+^ ECL signals. The system based on alternating current (AC) electroosmotic flow achieves an LOD of 2 fg mL^−1^ for cTnI in 5 min and 10 aM for miRNAs in 10 min at room temperature. The point‐of‐care testing (POCT) device demonstrated 100% sensitivity and 98% specificity for cTnI detection in 123 clinical serum samples. For miR‐499‐5p, it exhibited 100% sensitivity and 97% specificity in 55 clinical serum samples. Continuous monitoring of these biomarkers in rats' saliva, urine, and interstitial fluid samples for 48 hours revealed observations rarely documented in biotic fluids. The ECL‐M POCT device stands as a top‐performing system for ECL analysis, offering immense potential for ultrasensitive, rapid, highly accurate, and facile detection and monitoring of acute diseases in POC settings.

## Graphical abstract

Fabrication of an alternating current electroosmotic flow‐based ultrasensitive electrochemiluminescence microfluidic (ECL‐M) system for ultrafast monitoring of proteins/miRNAs within ten minutes in saliva, urine, interstitial fluid, and serum samples. This washing‐free ECL‐M device allows effective detection of protein and miRNA biomarkers for acute myocardial infarction diagnosis, which provides a powerful tool for POCT testing within hospitals especially in point‐of‐care settings.

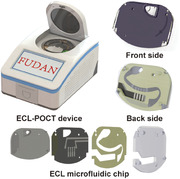

## 1 Introduction

Electrochemiluminescence (ECL) integrates the advantages of both chemiluminescence and electrochemistry, including high sensitivity, high stability, low background, and wide dynamic range.^[^ ^1^ ^]^ Therefore, it has been widely adopted in bioanalysis and clinical diagnosis.^[^ ^2^ ^]^ Commercial ECL devices such as Roche Cobas e601 have the advantage of high accuracy, throughput, and automation for the detection of tumor markers and infectious disease via biotin‐streptavidin recognition.^[^ ^3^ ^]^ Unfortunately, commercial ECL detection of serological and nucleic acid faces the challenge of a complex process, importability, and long preprocessing time. Moreover, the sensitivity of traditional ECL detection does not meet the requirement of analyzing biomarkers at ultralow abundance in untreated non‐blood samples.^[^ ^4^ ^]^ Therefore, an ultrasensitive, low‐cost, miniaturized, and rapid ECL platform is in great need of disease in health centers. To overcome these difficulties, microfluidic chips are considered to integrate with ECL method. Microfluidics, as automatic manipulation devices, possess micropumps, microchannels, and functional chambers.^[^ ^5^ ^]^ Microfluidics has the advantages of automated operation, less reagent consumption, high throughput, less contamination, etc.^[^ ^6^ ^]^ Owing to the bottlenecks of lab‐based ECL methods and increasing demand for real time on‐site diagnosis, the newly developed user friendly, ECL‐microfluidic (ECL‐M) POCT device should be developed to satisfy the need of clinical diagnosis. Up until now, ECL‐M has been paying increasing attention to POCT devices. Most ECL‐M POCT devices are based on 3D paper‐based microfluidics, which are low‐cost and disposable.^[^ ^7^ ^]^ While the results are often recorded by CCD camera or large ECL workstation.^[^ ^8^ ^]^ Recently, the portable power sources, wireless ECL devices, and bipolar electrodes have emerged in ECL systems.^[^ ^9^ ^]^ For instance, Qi et al. firstly developed a wireless ECL mini device.^[^ ^10^ ^]^ A high throughput, screening platform was well established via a wireless energy transmission strategy and results were recorded by CCD. It has great potential for developing portable ECL‐M screening devices. However, the inability of intensity‐based ECL‐M methods to achieve detection to single‐molecule level and the need for long incubation time to reach detectable levels have limited the further development of ECL‐M. Therefore, an automatic, portable, intensity‐based ECL‐M for disease diagnostics is still in high demand.

Due to low abundance of many acute disease‐relevant biomarkers in body fluid samples, serum sample was considered useful for diagnosis at hospital, as well as for monitoring the level of morbidity.^[^ ^11^ ^]^ For example, acute myocardial infarction (AMI) is an acute myocardial necrosis due to the persistent and severe ischemia, which leads to millions of deaths around the world annually.^[^ ^12^ ^]^ Cardiac troponin I (cTnI) and miR‐499‐5p are the eminent biomarkers for myocardial injury diagnosis.^[^ ^13^ ^]^ Cardiac troponin I plays a significant role as a molecular switch mediating calcium regulation in the contraction of skeletal and cardiac muscle.^[^ ^14^ ^]^ The concentration of cTnI could reflect the degree of myocardial injury with strong specificity for AMI and high diagnostic sensitivity.^[^ ^15^ ^]^ Furthermore, miR‐499‐5p has been shown to be expressed in myocardium and skeletal muscle in mammals. Previous studies have shown that miR‐499‐5p is highly expressed in the blood of AMI patients.^[^ ^16^ ^]^ Cardiac troponin I (cTnI) and miR‐499‐5p are considered prominent biomarkers due to the high sensitivity.^[^ ^17^ ^]^ However, the expression levels of cTnI increase in peripheral blood samples at 3−6 h post AMI, and peaks at 12−24 h, which exhibits no symptoms for AMI diagnosis within the first 2 h. For those with acute disease in need of timely diagnosis, detecting biomarkers in blood samples sometimes is not satisfactory. Therefore, it is urgent to develop a sensitive, portable, and rapid device that is capable to analyze ultra‐low levels of potential biomarkers in easily obtained clinical samples such as saliva, urine, and interstitial fluid, which include various biomarkers (e.g., proteins and nucleic acids) closely related to blood concentration.^[^ ^11^, ^18^ ^]^ To solve the problems, applying an alternating current voltage (AC voltage) to ECL‐M chips has become popular among researchers for rapid and ultrasensitive manipulation of the targets such as biomolecules and miRNAs.^[^ ^19^ ^]^ Electric field at a low voltage is capable to drive the motion of charged analytes, which increases the speed of tests and capturing efficiency in whole blood, plasma, or serum.^[^ ^20^ ^]^ Therefore, the sensitivity upgrades and the whole turnaround time decrease simultaneously. Moreover, bodily fluid is an attractive biospecimen mainly due to the ease of collection and massive acquisition in human body for POCT test.^[^ ^21^ ^]^

In this article, we describe an ultrasensitive, rapid, and low‐cost AC‐driven ECL‐M POCT device for monitoring and detection of cTnI and miR‐499‐5p in unprocessed saliva, urine, and interstitial fluid samples. Integrating AC voltage with sandwich immune system and surface plasmon coupling method, the cTnI with LOD of 2 fg mL^−1^ (within 5 min) and miR‐499‐5p with LOD of 10 aM (within 10 min at room temperature) are capable to be tested from unprocessed samples, even in bodily fluid. The proposed ECL‐M POCT device enables rapid and ultrasensitive detection of proteins and nucleic acids in biofluids for acute disease diagnosis. Moreover, ECL‐M POCT device makes it possible to monitor the time‐course of both protein and miRNA biomarker levels in bodily fluids, thus providing a powerful tool for POCT testing within hospitals especially in point‐of‐care settings.

## 2 Results and Discussion

### 2.1 Design and Construction of the ECL‐M POCT Device and ECL‐M Chip

**Figure 1:**
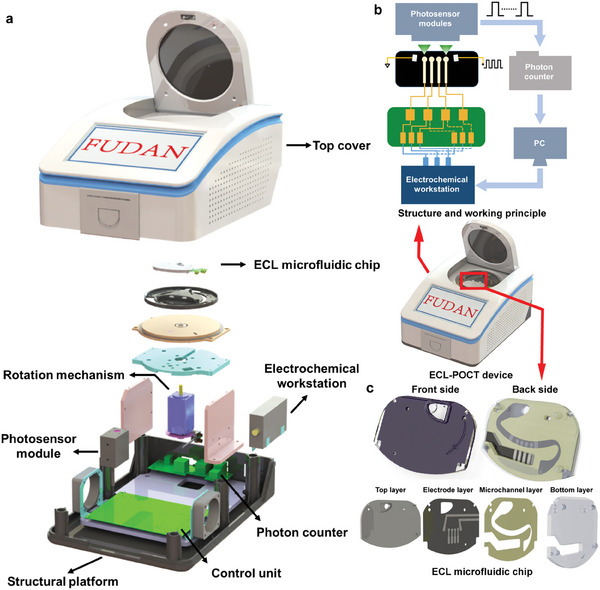
a) Overview of the ECL‐M POCT system. The homemade ECL‐M POCT device contains a structure platform, an electrochemical workstation, a photosensor module, a photon counter, ECL‐M chip, rotation mechanism, and a PC control unit. b) Structure and working principle of the ECL‐M POCT device. c) Overview of the ECL‐M chip (front side and back side) and an exploded view of the ECL microfluidic chip, which includes top layer, electrode layer, microchannel layer and bottom layer.

The ECL‐M POCT device is an automatic system that supports three parallel measurements (**Figure** **1**). The ECL‐M device (length × width × height, 282 mm × 215 mm × 135 mm) (Figure S1, Supporting Information) mainly consists of an electrochemical workstation, a photosensor module, a photon counter, three microfluidic chips, a rotation motor, and a PC control unit (Figure 1a). The photosensor module converts light signal to electrical pulse. The photon counter counters the number of the photon and outputs the result (The photon counting value is defined as ECL intensity for further analysis). The rotation motor is responsible for moving the microfluidic chip to the detection position, and the PC control unit can control and monitor each module. The power of the proposed ECL‐M POCT device can be provided via either a 220 V AC to 12 V DC adaptor or a lithium battery. The workflow of the ECL‐POCT device is as follows (Figure 1b): Once the ECL process exists, the rotation motor drives the ECL‐M chip to the detection position. The electrochemical workstation applies the cyclic voltammetry to the designated WE (WE_1_ or WE_2_), RE, CE on the ECL microfluidic chip, which electrochemically generates considerable excited states of luminophores and then relaxes to the ground states with light emission. The light signal is captured and converted to the electrical pulse by the photosensor module. Later, the number of the electrical pulse is countered by the photon counter, and the result of photon number is exported in the form of pulse numbers. At the end of the first test, the ECL microfluidic chip automatically turns to the next test until three microfluidic chips are tested. Owing to the low cost of the chip, the estimated material cost for an ECL‐M chip capable of testing two samples is about US$ 0.15. Furthermore, we have collected the cost prices of cTnI detection kits in the domestic market and found that our ECL‐M chip prices are significantly lower than those in the domestic market (Table S2, Supporting Information). Considering the cost for POCT devices, our proposed ECL‐M POCT device costs roughly US$ 3383 (Table S3, Supporting Information), with the bulk of cost from the PMT (Hamamatsu Photonics H10682‐210, US$ 1528), the photon counting unit (Hamamatsu Photonics CH297‐011, US$ 170), the electrochemical workstation (Wuhan Meoguan Biotechnology EC‐1550A, US$ 420), Control unit (homemade, US$ 170), Rotation mechanism (Oriental motor PKP525N12B‐L, US$ 165) and Structure platform (homemade, US$ 500). In addition, the cost prices of chemiluminescence detection systems in the domestic market were also collected, including Shine i1900 (Increcare, US$ 4980), MAGLUMI X3 (Snibe Diagnostic, US$ 15 000) and CL‐900i (Mindray, US$ 22 000), which were much higher than the cost of ECL‐M system (Table S4, Supporting Information). The lower cost of the ECL‐M system would facilitate the promotion of AMI biomarker detection and effectively prevent the occurrence of AMI.

The designed microfluidic chip (length × width × height, 55 mm × 35 mm × 5.2 mm) mainly consists of four parts including top layer, electrode layer, microchannel layer, and bottom layer (Figure 1c; Figure S2,S3, Supporting Information). The top layer and the bottom layer are made of polycarbonate (PC), which provide the injection port and the blowhole. The single‐sided electrode layer is made of opaque polyvinyl chloride (PVC) to prevent high background signals from visible light. Moreover, it contains six screen‐printing electrodes including two Ag electrodes to produce AC electric field, working electrode 1 (WE_1_), reference electrode (RE), counter electrode (CE), and working electrode 2 (WE_2_). The microchannel layer consists of 0.2 mm‐thick double‐sided adhesive to form a flow channel. The reaction liquid is supposed to be injected from the beginning and fill the entire channel without outflow, which reduces the environmental pollution of reagents. The assembly of the ECL microfluidic chip is as follows: The electrode layer is firstly attached to the top layer, then the microchannel layer is stuck above. Finally, the top layer complex and bottom layer are stuck together via microchannel layer and mechanical pressure. Due to the irreversibility of reactions on the working electrodes and complex chip assembly process using tablet press machine, the ECL‐M chip containing WE_1_ and WE_2_ is disposable.

### 2.2 Schematic Illustration and Characterization of ECL‐M POCT System

**Figure 2:**
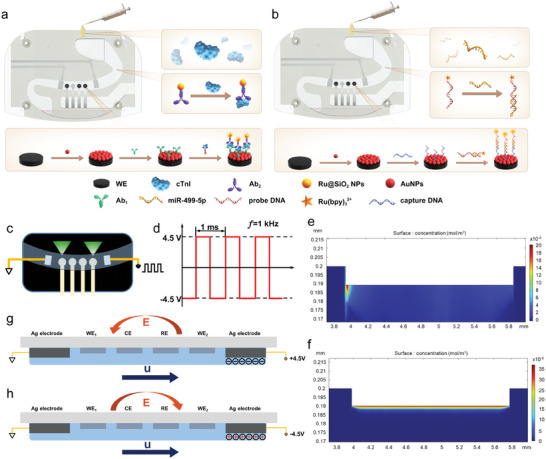
a) Detection procedure of ECL‐M POCT device for biomolecule. b) Detection procedure of ECL‐M POCT device for miRNA. c) Schematic illustrations of AC voltage‐driven ECL‐M POCT system. The AC voltage was applied to the two Ag electrodes with a form of square wave. The electric field was produced in the flow channel between the two Ag electrodes. d) Schematic diagram of the AC square wave signal. Capturing conditions: V_pp_ = 9 V and f_AC_ = 1 kHz. e), f) Concentration field distribution of cTnI antigen under diffusion process (e) and with AC‐driven electrical field (f). g), h) Schematic diagram of ACEO mechanism in this ECL‐M POCT system under the voltage of −4.5 V (g) and +4.5 V (h).

The unique advantage of ECL‐M POCT device and the AC‐voltage driven preprocessing strategy of ECL‐M chip enable ultrasensitive and rapid detection of proteins and miRNA in bulk biofluids (**Figure** **2**). In this study, detection of cTnI was performed on WE_1_ based on sandwich immune system (Figure 2a). AuNPs were modified on WE_1_ to support considerable Ab_1_ owing to the high surface area of AuNPs.^[^ ^22^ ^]^ Then Ab_1_ was attached to the WE_1_ via covalent bond with AuNPs. When the protein target was added into the ECL‐M chip, Ru@SiO_2_ NPs‐Ab_2_ complex specifically binds the analyte and then flowed to the WE_1_ until it was captured by the immobilized Ab_1_. Detection of miR‐499‐5p was undergone on WE_2_ based on surface plasmon resonance (SPR) strategy (Figure 2b).^[^ ^23^ ^]^ First, AuNPs were modified on WE_2_ and connected to thiolated capture DNA. When the miRNA analyte was injected into the ECL‐M chip, the complementation of bases occurred between Ru(bpy)_3_^2+^/probe DNA and miRNA. Later, the complex automatically flowed to WE_2_ and complemented with the base of capture DNA. The capture DNA chain could regulate the distance between Ru(bpy)_3_^2+^ and AuNPs, which obtained obvious ECL signals. AC voltage with the square wave was synchronous to accelerate the speed of analyte and improve the capture efficiency (Figure 2c,d; Figure S4, Supporting Information). COMSOL simulations illustrated the electrical field distribution in the ECL‐M chip under the voltage of −4.5 V and +4.5 V (Figure S5, Supporting Information). The uniform sphere morphologies of AuNPs and Ru@SiO_2_ NPs were characterized by TEM (Figure S6, Supporting Information). The sizes of two nanoparticles were then measured by DLS as the diameter of 24.36 nm and 50.75 nm, respectively (Figure S7, Supporting Information). After binding with Ab_2_, the diameter of Ru@SiO_2_ NPs‐Ab_2_ was 68.06 nm, indicating successful modification of Ab_2_ on Ru@SiO_2_ NPs. In the UV‐vis absorption spectrum (Figure S8, Supporting Information), AuNPs exhibited a characteristic absorption peak at 520 nm.^[^ ^22^ ^]^ Ru(bpy)_3_^2+^ displayed two characteristic absorption peaks at 285 nm and 462 nm, while two main absorption peaks of Ru@SiO_2_ NPs matched those of the absorbance, indicating the successful existence of Ru(bpy)_3_^2+^ into SiO_2_.^[^ ^24^ ^]^ The UV‐vis spectrum of AuNPs exhibited great overlap with the ECL emission peak of Ru(bpy)_3_^2+^ at 620 nm.^[^ ^25^ ^]^ AuNPs could stimulate adjacent electromagnetic field by excited luminophore species, leading to the enhancement of excitation rate and emission efficiency of the luminophore.^[^ ^26^ ^]^ Therefore, the ECL intensity of Ru(bpy)_3_^2+^ was enhanced. Zeta potentials of Ru(bpy)_3_^2+^, Ru@SiO_2_ NPs, Ru@SiO_2_‐NH_2_, AuNPs were characterized (Figure S9, Supporting Information). Ru@SiO_2_ NPs (−10.23 ± 0.38 mV) exhibited negative charge due to the silanol group outside Ru(bpy)_3_^2+^ (72.10 ± 0.70 mV). The negative charge of Ru@SiO_2_ NPs changed to 2.15 ± 0.01 mV after amination, demonstrating successful modification of amino groups on Ru@SiO_2_ NPs. After binding with Ab_2_, Ru@SiO_2_ NPs‐Ab_2_ complex displayed positive charge of 5.257 ± 0.18 mV mainly due to the modification of Ab_2_ on Ru@SiO_2_ NPs.^[^ ^27^ ^]^ AuNPs showed −25.20 ± 0.66 mV. Atomic force microscopy (AFM) images exhibited the successful immobilization of Ab_1_ on gold modified electrode and no aggregation was observed (Figure S10, Supporting Information). The loading efficiency of Ab_1_ was estimated as 31.7%, along with that of capture DNA on AuNPs was 92.4% (Figure S11, Supporting Information). The spectrum of X‐ray photoelectron spectroscopy (XPS) N 1s peak (Figure S12, Supporting Information) showed the immobilization of Ab_1_ on gold‐modified electrode. Micrograph of two liquid‐phase states (dyed yellow and blue) illustrated the successful separation by engineered fluid, which greatly avoided the cross‐contamination and non‐specific signal (Figure S13, Supporting Information).

### 2.3 Enhancement Mechanism of AC‐driven ECL‐M Sensitivity

Compared with conventional ECL method, the proposed AC‐driven ECL‐M POCT chip has the obvious advantage of effective capture of charged biomolecules such as antibodies or nucleic acids. The ECL signal of blank showed almost the same at very low background signals with and without AC‐driven electric field, while 10 pg mL^−1^ cTnI showed 2.6‐fold enhancement under AC‐driven electric field in five minutes (Figure S14, Supporting Information). This was mainly attributed to the existence of high‐intensity asymmetric electric field provided by the AC driving electrode which greatly promoted rapid immune analysis. It was obvious that the analysis under AC‐driven field led to excellent enhancement of ECL signals. Though DC electroosmotic micropump has the advantages of low manufacturing cost, and simple design, making it an effective way to drive liquids, it generates electrolytic reactions of the solution, massive electric heat, and bubbles in the reaction system, which do harm to the stability of microfluidic flow. COMSOL simulation was adopted by Commercial Software (COMSOL Multiphysics) to explore and calculate the enhancement mechanism of AC‐driven ECL‐M sensitivity. The COMSOL simulation model and boundary of the flow field were established following the dimensions of the electrodes and microchannels (Figure S15, Supporting Information). The related parameters of COMSOL simulation were listed in Table S5 (Supporting Information).

The fluid in the ECL‐M chamber maintains the laminar flow state and diffusion is the mass transfer process without being disturbed by the electrical field. And the diffusion velocity u satisfies the Equation (1)

(1) u=D/h

where D and h refer to diffusion coefficient and channel height, respectively. D was generally 2.120 × 10^−9^ m^2^ s^−1^ while h was 2 × 10^−4^ m.^[^ ^28^ ^]^ The diffusion velocity was calculated as about 10.60 µm  s^−1^. The antigen concentration satisfies the Equation (2)

(2) $$\frac{\partial c}{\partial t}=\nabla^{2}\;c$$

During the diffusion process, antigen was gradually captured by antibody via specific recognition (Figure 2e; Table S6, Figure S16, Supporting Information). The maximum local concentration of antigen captured by the modified electrode surface was calculated as 2.05134 × 10^−7^ M.

Three mechanisms including capillary electrophoresis, AC electrothermal (ACET), and AC electroosmosis (ACEO) for the fluid flow are considered under AC‐driven electric field in the proposed ECL‐M chip. The migration rate of charged substances in capillary electrophoresis follows the Equation (3)

(3) $$V=\frac{qE}{6\pi r\eta }$$

where charge numbers (q) were referred as 14 net charges, electric potential (*E*) was estimated as 346.15 V/m, particle radius (*r*) was 21.65 nm, solution viscosity (η) was calculated as 1.53 Cp (Figure S17, Supporting Information). V was calculated as 1.2418 µm s−1. Proteins can be positively or negatively charged depending on the relationship between isoelectric point (pI) of the proteins and pH value of the electrolyte. Owing to the pI of cTnI antigen being 9.87,^[^ ^29^ ^]^ the cTnI antigen is positively charged in PBS solution (pH 7.4).

Assuming that the system is in a medium with uniform electrical properties, the electric potential is obtained by the DC Laplace Equation (4)

(4) $$\nabla^{2}\phi =0,\;\vec{E}=-\nabla$$

The temperature gradient, AC force is obtained via the Equation (5), (6) below

(5) $$k\nabla^{2}T+\sigma \;{\left|\vec{E}\right|}^{2}=0$$

(6) $$\langle \vec{f_{e}}\rangle =\frac{1}{2}\cdot \frac{\varepsilon \left(\alpha -\beta \right)}{1+\left(2\pi f\varepsilon /\sigma \right)}\left(\nabla T\cdot \vec{E}\right)\vec{E}-\frac{1}{4}\varepsilon \alpha {\left|\vec{E}\right|}^{2}\nabla T$$

where k, T, f_e_, α, β were media thermal conductivity, temperature, Electrical heat, rate of capacitance changes with temperature, rate of conductivity changes with temperature, respectively.

The fluid velocity in ECL‐M channel satisfies Navier‐Stokes equation and continuity Equation (7)

(7) $$-\nabla p+\mu \nabla^{2}\vec{u}+\langle \vec{f_{e}}\rangle =0,\;\;\nabla \cdot \vec{u}=0$$

The maximum temperature in ACET model was calculated as 3.53 °C, which was the difference between the maximum temperature of 301.680 K and the initial temperature of 298.15 K (Figure S18, Supporting Information). Slight temperature rise was observed in this system, indicating negligible effect of ACET. The maximum local velocity for ACET was calculated as 4.734 × 10^−4^ µm s^−1^ (Figure S19, Supporting Information).

The relaxation frequency equation for electrode polarization was calculated employing the Equation (8)

(8) f=σ/ε

where σ refers to conductivity and ε refers to dielectric constant. The σ of antigen solution was measured as 38.1 mS cm^−1^ and ε was calculated as 7.08 × 10^−10^ (Figure S20, Supporting Information). The relaxation frequency was calculated as 1.98 × 10^5^ kHz, which was far more than 1 kHz in this experiment. Therefore, the fluid flow was dominated by ACEO in the proposed AC‐driven ECL‐M channel. The fluid in ECL‐M channel satisfies Navier‐Stokes Equation (9)

(9) $$\rho \;\frac{\partial u}{\partial t}=\nabla^{2}\;u-\nabla p+f_{e}$$

(10) ∇·u=0

(11) $$f_{e}=\rho E-\frac{1}{2}\;E^{2}-\nabla \varepsilon =-\varepsilon \frac{\nabla \sigma \cdot \nabla \phi \;}{\sigma }\nabla \phi -\frac{1}{2}{\left(\nabla \phi \right)}^{2}\cdot \nabla \varepsilon$$

Where ρ, u, η, p were density, viscosity, velocity, and pressure of the fluid, respectively.

The instantaneous ACEO flow velocity follows the Equation (12)

(12) $$u=\varepsilon_{0}\;\varepsilon_{r}\frac{E_{t}\zeta }{\eta }$$

where *E_t_*,ζ refer to the tangential component of the electric field and zeta potential, respectively. Under the influence of ACEO field, the concentration of antigen was following the Equation (13)

(13) $$\frac{\partial c}{\partial t}+u_{ac}\nabla c=\nabla^{2}\;c$$

The maximum local concentration of antigen captured by the modified electrode surface was calculated as 3.619 × 10^−7^ M, which was about 1.76 times that without AC‐driven electrical field (Figure 2f). Moreover, compared with the diffusion velocity (10.60 µm s^−1^), capillary electrophoresis (1.2418 µm s^−1^), and ACET effect (4.734 × 10^−4^ µm s^−1^) of antigen solution, the maximum local velocity for ACEO was calculated as 1711.224 µm s^−1^ (Figure S21, Supporting Information), which was the main factor in AC kinetic effect (almost 155‐fold enhancement than that in diffusion process). When the left driving electrode was grounded and the right driving electrode was +4.5 V, the electric field direction was from right to the left (Figure 2g). Massive negative charge ions in the double electric layer were then accumulated on the right driving electrode, which led to the opposite movement direction of fluid nearby to the direction of the electric field. Therefore, the net flow direction of the fluid is from left to right, driving the solution containing antigen‐Ab_2_ complexes captured by Ab_1_ to flow forward and be captured. When the left driving electrode was grounded and the right driving electrode was changed to −4.5 V, the double electric layer near the right electrode was accumulated with positively charged ions, and the net flow direction of the fluid remains from left to right (Figure 2h). Although the electric field direction changed under the application of AC voltage, it remained the same flow direction of fluid from left to the right, which was the constant driving force of pumping antigen‐Ab_2_ complexes to the Ab_1_ modified working electrode (Movie S1, Supporting Information). It was obvious that the speed of immunoassay under the AC‐driven electric field in the ECL‐M chip was much faster than the natural diffusion rate, which contributed to the enhancement of sensitivity and saving of time cost.

### 2.4 Ultrasensitive Bio‐Detection by AC‐Driven ECL‐M Device

**Figure 3:**
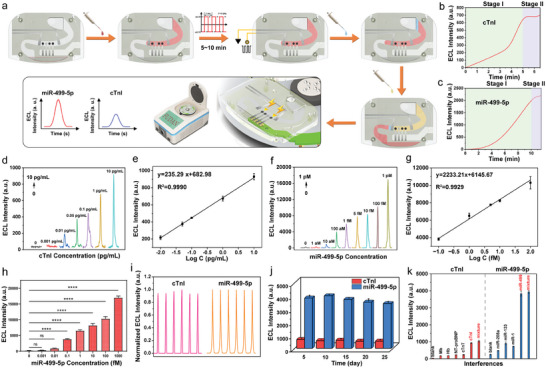
a) Workflow of AC voltage‐driven ECL‐M POCT device. b), c) Real‐time monitoring plots of the reaction process in the entire microfluidics system for detection of cTnI (b) and miR‐499‐5p (c). Stage I refers to the incubation period of targets under AC‐driven field. Stage II refers to the stable ECL detection period under electrical excitation, respectively. d) ECL signals of the proposed ECL‐M biosensor under different concentrations of cTnI varying from 0, 0.001, 0.01, 0.05, 0.1, 1, 10 pg mL^−1^, respectively. e) Calibration plots of the ECL intensity vs the logarithm of cTnI concentration. Error bars are determined by the standard deviation of 3 parallel measurements. f) ECL signals of the proposed ECL‐M biosensor under different concentrations of miR‐499‐5p varying from 0, 1 aM, 10 aM, 100 aM, 1 fM, 5 fM, 10 fM, 100 fM, 1 pM, respectively. g) Calibration plots of the ECL intensity vs the logarithm of miR‐499‐5p concentration. Error bars are determined by the standard deviation of 3 parallel measurements. h) Significant difference between ECL signal of different miR‐499‐5p concentration and blank. *p < 0.05, **p < 0.01, ***p < 0.001, ****p < 0.0001 vs. Blank; n.s., no significant. i) Normalized stability of the proposed ECL‐M biosensor incubated with 1 pg mL^−1^ cTnI and 100 aM miR‐499‐5p under consecutive cyclic potential scans for 6 cycles, respectively. j) Reproducibility of the proposed ECL‐M biosensor incubated with 1 pg mL^−1^ cTnI and 100 aM miR‐499‐5p. The ECL experiment was carried out in ECL working solution containing 0.1 M TPrA under the voltage of 0 to +2.0 V. Scan rate was 100 mV s^−1^. k) Selectivity of the ECL‐M chip with different interferences. For protein selectivity validation: Mb (1 ng mL^−1^), Hb (1 ng mL^−1^), NT‐proBNP (1 ng mL^−1^), cTnT (1 ng mL^−1^), cTnI (10 pg mL^−1^), and a mixture (Mb + Hb + NT‐proBNP + cTnT + cTnI).

The incubation solution containing analytes was first added and ran along the microfluidic channel to the second drive electrode (**Figure** **3**a). The AC voltage was applied to drive the charged biomolecule back and forth along the microfluidic channel. After a period of incubation time, analytes were combined with the corresponding modified electrodes. Then 2 µL of engineered fluid which was electronic fluoride solution (incompatible with water) was injected into the ECL‐M chip before the addition of ECL working solution to prevent the ECL signal interferences from the unbound Ru@SiO_2_ NPs and Ru(bpy)_3_^2+^. Then the ECL working solution containing TPrA was injected to stimulate ECL and generate ECL signals on WEs. Three separated ECL‐M chips could be tested automatically and simultaneously through the rotation motor of the ECL‐M POCT device and the whole turnaround time for three tests took about 6.5 min (5 min for simultaneous incubation and 90 s for detection of cTnI three times) and 11.5 min (10 min for simultaneous incubation and 90 s for detection of miR‐499‐5p three times) at room temperature. The real‐time monitoring plots for detection of cTnI and miR‐499‐5p during the entire ECL‐M system were divided into two regions (Figure 3b,c): (I) Stage I (5 min < t < 10 min), where analytes were incubated under AC‐driven field; (II) Stage II (t = 1.5 min), where the stable ECL signal response under electrical excitation. The proposed ECL‐M device enabled us to test cTnI and miR‐499‐5p rapidly under mild conditions without manual operation. To validate the successful step‐by‐step modification of the ECL‐M biosensor for sensitive and rapid detection of cTnI and miR‐499‐5p, 5 mM [Fe (CN)_6_]^3‐/4−^ solution containing 0.1 M KCl was utilized for EIS characterization (Figure S22, Supporting Information). In comparison to that of the bare WE (Figure S22a, Supporting Information), AuNPs modified electrode showed a decrease of the semicircle diameter owing to the good conductivity of AuNPs (Figure S22b, Supporting Information). Ab_1_, cTnI, Ru@SiO_2_ NPs‐Ab_2_ conjugates and capture DNA, miR‐499‐5p led to the successive increase of the semicircle diameter due to the hinderance effect of electron transfer by macromolecules and repulsive force between [Fe (CN)_6_]^3‐/4−^ and nucleic acids (Figure S22c,d,e,f,g, Supporting Information). The semicircle diameter decreased under the existence of Ru(bpy)_3_^2+^/probe DNA due to the interaction between [Fe (CN)_6_]^3‐/4−^ and positively charged Ru(bpy)_3_^2+^ (Figure S22h, Supporting Information). When AC voltage was applied, the oxidation current was observed higher than that without AC voltage, probably due to the AC voltage led to the easier oxidation of luminophores, thus causing an increase in the excited species of Ru@SiO_2_ NPs (Figure S23, Supporting Information).

Under optimal conditions (Figure S24,S25, Supporting Information), the sensitivity and quantitative ability of this ECL‐microfluidic system were comprehensively evaluated in detecting cTnI and miR‐499‐5p. A series of concentrations of cTnI ranging from 0 to 10 pg mL^−1^ were measured (Figure 3e). From the results, we observed that with the increase of cTnI concentration, the photon number continued to increase. The ECL intensity was linearly related to the logarithms of cTnI concentration (pg/mL, *C*) (Figure 3f) following the equation as ECL Intensity = 682.98 + 235.29 log *C*_cTnI_ (R^2^ = 0.9990) with a LOD of 2 fg mL^−1^. Similarly, the ECL intensity also increased corresponding to the increase of miR‐499‐5p ranging from 0 to 1 pM (Figure 3g). A linear relationship was observed between ECL intensity and miR‐499‐5p concentration (fM, *C*) in the range of 0.1–100 fM following the equation as ECL Intensity = 6145.67 + 2233.21 log*C*_miR‐499‐5p_ (R^2^ = 0.9929) with the LOD of 10 aM (Figure 3h). The LOD was calculated as 3δ/k, where δ represents the standard deviation of blank signals (n = 3) and k is the slope of the linear curve. Near the LOD of 10 aM, 4 out of 5 ECL‐M chips showed responses (Figure 3i). Statistical analysis exhibited significant difference of 0.1‐1000 fM versus blank (****p < 0.0001).

The stability of ECL‐M biosensors for detection of cTnI (1 pg mL^−1^) and miR‐499‐5p (100 aM) were evaluated by continuous scanning for 6 cycles each (Figure 3j). Stable ECL signals were obtained with the RSD of 1.92% for cTnI and 0.37% for miR‐499‐5p, demonstrating acceptable stability of this ECL‐M POCT platform. The reproducibility of the ECL‐M system was estimated three times under the same conditions every 5 days for 25 days (Figure 3h). The relative standard deviation (RSD) was calculated as 8.10% with cTnI concentration of 1 pg mL^−1^ and 5.80% with miR‐499‐5p concentration of 100 aM, indicating good reproducibility of this system within 25 days.

Selectivity tests for cTnI and miR‐499‐5p were carried out individually. Several protein interferences such as myoglobin (Mb), hemoglobin (Hb), N‐terminal pro‐B‐type natriuretic peptide (NT‐proBNP), cardiac troponin T (cTnT) for cTnI selectivity validation, while several miRNA interferences such as miR‐208a, miR‐133, miR‐1 for miR‐499‐5p selectivity validation were considered independently (Figure 3k). The as‐fabricated platform with these interferences exhibited similar response as blank signals. Moreover, less than a 6.3% difference with the cTnI solution compared to those of cTnI with interferences, along with a 2.7% difference for miR‐499‐5p with the miR‐499‐5p solution compared to the mixture were obtained, demonstrating that the constructed ECL‐M POCT platform possessed good selectivity for the detection of cTnI and miR‐499‐5p.

### 2.5 Direct Clinical Diagnosis of cTnI and miR‐499‐5p in Untreated Serum Samples

**Figure 4:**
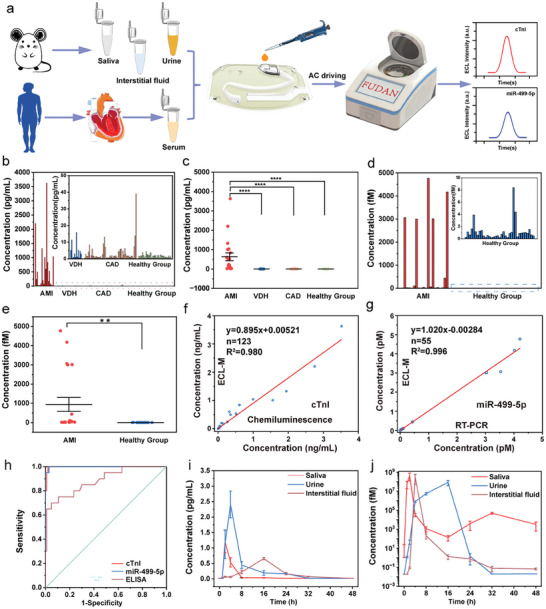
a) Workflows for protein and nucleic acid testing in post AMI diagnosis and monitoring by ECL‐M POCT device. b) Concentration of cTnI corresponding to patients with AMI, valvular disease of the heart (VDH), coronary artery disease (CAD), and in healthy group. c) Concentration levels of cTnI in patients with AMI, VDH, CAD, and healthy group (****p < 0.0001; One‐way ANOVA; AMI, n = 20, VDH, n = 12, CAD, n = 56, Healthy Group, n = 35). d) Concentration of miR‐499‐5p corresponding to patients with AMI and healthy group. e) Concentration levels of miR‐499‐5p in patients with AMI and in healthy group (**p < 0.01; unpaired t‐test; AMI, n = 20, Healthy Group, n = 35). f), g) Correlation between the results using ECL‐M system and the real concentration from hospital of cTnI f) and miR‐499‐5p g). The concentration unit were pg mL^−1^ (f) and fM (g). h) Receiver operating curve (ROC) curves of the ECL‐M POCT system for the detection of cTnI and miR‐499‐5p, ELISA method for the detection of cTnI in AMI diagnosis from clinical samples. i), j) The time course of cTnI (i) and miR‐499‐5p (j) levels from saliva, urine and interstitial fluid samples of AMI vs. Sham rats at 0, 1, 2, 4, 8, 16, 24, 32, and 48 h post‐surgery. *p<0.05 **p < 0.01, ***p < 0.001, ****p < 0.0001; n. s., no significant vs. Sham rats at the same time point; Two‐way ANOVA; n=8 animals per group per time point.

We have applied ECL‐M POCT device to directly detect cTnI and miR‐499‐5p in untreated human serum and monitored the concentration levels of cTnI and miR‐499‐5p with time course in AMI diagnosis from bodily fluid samples in rats (**Figure** **4**a). As shown in Figure S26 (Supporting Information), The ECL signals towards cTnI (concentration of 10 pg mL^−1^) and miR‐499‐5p (concentration of 100 fM) showed no significant difference in PBS (pH 7.4), artificial saliva (pH 6.8), urine (pH 5.7), interstitial fluid samples (pH 7.4), and serum, demonstrating almost no interferences of other components in the test samples. Human clinical serum samples were obtained from the First Affiliated Hospital of Xi'an Jiaotong University and Yizheng Hospital of Traditional Chinese Medicine for biomolecule and nucleic acid analysis (Table S8, Supporting Information). The human research was approved by the Ethics Committee of Fudan University and complied with all relevant ethical regulations (IRB No. FE22185R). Informed consent was given by human participants, and they were compensated. cTnI and miR‐499‐5p could be detected directly by the ECL‐M POCT device (Figure 4b,d). We compared the diagnostics value of cTnI in AMI within other heart‐related diseases (such as VDH and CAD) and also in healthy group (Figure 4c). The significant difference (****p < 0.0001) between AMI and VDH, CAD, healthy group was exhibited, confirming that cTnI was idiosyncratic to AMI diagnosis. Noteworthily, previous works have demonstrated the expression level of miR‐499‐5p was positively related to other heart‐related disease.^[^ ^30^ ^]^ Therefore, the diagnostics value of miR‐499‐5p in AMI was compared with in healthy group, which exhibited significant differences (**p < 0.01) between AMI and healthy group (Figure 4e).^[^ ^31^ ^]^ The correlation between the real concentration obtained from hospital and the tested concentration for two biomarkers (Figure 4f,g). The concentration of cTnI using chemiluminescence in hospital showed good consistency with the tested concentration using ECL‐M POCT system (y = 0.895x + 5.21, R^2^ = 0.980), while the concentration of miR‐499‐5p using RT‐PCR in hospital showed good consistency with tested using ECL‐M POCT system (y = 1.02x–2.84, R^2^ = 0.996). ROC curves of cTnI and miR‐499‐5p using our ECL‐M POCT device were compared with that of cTnI using ELISA kit via human clinical serum samples (Figure 4h). The diagnostic efficacy of the proposed device was competitive in both protein (AUC_ROC_ was 0.998; the Youden index (J) was 0.9806) and nucleic acid (AUC_ROC_ was 0.999; the Youden index (J) was 0.9714). Based on the defined cutoff value (14.20 pg mL^−1^) of cTnI for diagnosis of AMI, 20/20 (100% sensitivity) AMI positive samples and 101/103 (98% specificity) AMI negative samples were identified. For detection of miR‐499‐5p, 20/20 (100% sensitivity) AMI positive samples and 34/35 (97% specificity) AMI negative samples were classified with the cutoff value of 4.55 fM, illustrating that the results of detecting cTnI and miR‐499‐5p were highly consistent with high accuracy.^[^ ^32^ ^]^ Compared with cTnI testing using ELISA kit (AUC_ROC_ was 0.885; the Youden index (J) was 0.6515), our device exhibited higher accuracy than commercial ELISA kit.

### 2.6 Direct Real‐Time Monitoring of cTnI and miR‐499‐5p in Rat Models

The unprocessed bodily fluid samples were diluted and injected without time‐consuming purification, nucleic acid extraction, and amplification procedures. AMI was successfully surgically induced in rat models, and bodily fluid samples were obtained for cTnI and miR‐499‐5p detection (Figure S27, Supporting Information). The time‐course of cTnI (Figure 4i) and miR‐499‐5p (Figure 4j) levels accumulation in saliva, urine and interstitial fluid samples post‐AMI were firstly monitored. The peak of cTnI level in saliva appeared at 2 h post‐AMI, which was earlier than that in urine (4 h post‐AMI) and interstitial fluid (16 h post‐AMI) and returned to basal level by 8 h, indicating the potential value of early diagnosis via assessing cTnI levels in saliva samples in AMI diagnosis. While miR‐499‐5p in saliva peaked at 2 h post‐AMI, far earlier than that in interstitial fluid (4 h post‐AMI) and urine (16 h post‐AMI). The trend of cTnI and miR‐499‐5p at low abundance in bodily fluid was in good agreement within those serum samples, demonstrating great application potential of the proposed ultrasensitive and rapid ECL‐M device in on‐site POCT fields.

Compared with some state‐of‐the‐art cTnI detection methods, ECL‐M POCT devices have shown remarkable sensitivity and superior detection speed (Figure S28a, Table S9, Supporting Information). The limit of detection (LOD) of ECL‐M POCT method for cTnI was 2 fg mL^−1^, which was competitive to those of previous individual sensors based on ECL biosensor array (LOD: 0.79 pg mL^−1^),^[^ ^33^ ^]^ ECL immunoassay (LOD: 0.116 pg mL^−1^),^[^ ^34^ ^]^ In_2_O_3_ FET‐based biosensors (LOD: 1 pg mL^−1^),^[^ ^35^ ^]^ graphene‐based FET (LOD: 3.34 pg mL^−1^),^[^ ^36^ ^]^ Integrated photothermal‐pyroelectric biosensor (50 pg mL^−1^),^[^ ^37^ ^]^ dual‐signal ECL sensor (LOD: 3.39 fg mL^−1^).^[^ ^38^ ^]^ For general miRNA tests, high temperature and long incubation time are often required. The LOD of ECL‐M POCT method for miR‐499‐5p achieves 10 aM in 10 min at room temperature (10 aM, 10 min, 25 °C). Compared with other reported miRNA detection methods based on (poly A)‐based ECL sensor (5.1 fM, 65 min, 80 °C),^[^ ^39^ ^]^ SCRF biosensor (70.9 fM, 20 min, 80 °C),^[^ ^40^ ^]^ DNA tetrahedral nanostructure (10 fM, 300 min, 80 °C),^[^ ^41^ ^]^ 3C strategy (0.3 pM, 35 min, 95 °C),^[^ ^42^ ^]^ SNAzyme (0.3 pM, 300 min, 37 °C),^[^ ^43^ ^]^ SNAzyme (10 pM, 480 min, 37 °C)^[^ ^44^ ^]^ and G_4_/MOFzymes (1 fM, 10 min, 65 °C),^[^ ^45^ ^]^ our method showed potential advantages for portability, ultrahigh sensitivity, ultrafast detection and wide applicability (Figure S28b, Table S10, Supporting Information).

## 3 Conclusion

In summary, our ECL‐M POCT system based on AC electrokinetic effect has the advantages as follows: 1) reducing total turnaround time, sample volume and cost; 2) improving extraction and capturing efficiency via AC electrokinetic strategy; 3) reducing the difficulty of manual operation due to flexible switching of six detection points tested automatically; 4) allowing for ultrasensitive and rapid specific detection of proteins down to 2 fg mL^−1^ within 5 min and miRNAs down to 10 aM within 10 min at room temperature. 5) exhibiting excellent selectivity between AMI, other heart‐related disease such as VDH, CAD, and healthy group; 6) miR‐499‐5p was detectable in serum samples of healthy volunteers, which was ultralow or even undetectable in plasma from healthy group^[^ ^46^ ^]^; 7) first realizing the ultrasensitive and rapid quantification and monitoring of cTnI and miR‐499‐5p in untreated saliva, urine, and interstitial fluid; 8) cTnI and miR‐499‐5p in saliva were proved more effective (cTnI, peak at 2 h; miR‐499‐5p, peak at 2 h) than in serum samples(cTnI, peak at 12–24 h^[^ ^47^ ^]^; miR‐499‐5p, peak at 6–12 h^[^ ^48^ ^]^).

Our ECL‐M POCT system based on AC electrokinetic effect mainly focuses on urgent on‐site and point‐of‐care testing in army battlefields with portability, or in hospitals with lower funding budgets, or at home for patients with low professionalism. Further development of the ECL‐M POCT device is not just limited to heart‐related disease, with the sample types and sources also more diverse. It enables ultrasensitive joint diagnostics of multiple samples with an instrument automatic process within a very short time to further improve the diagnostic accuracy of the disease. In the future, our device may further improve the detection throughput, miniaturization, and equipment integration.

## 4 Experimental Section

### Materials and Instruments

MiR‐499‐5p, capture, probe DNA, miR‐208a, miR‐133, and miR‐1 were bought from Sangon Biological Co., Ltd. (Shanghai, China). The sequences of miRNAs were obtained from National Center for Biotechnology Information. Please see Table S1,S7 (Supporting Information) for utilized sequences. Engineered fluid (Novec 7500) was purchased from 3 M company (USA). N‐hydroxysuccinimide (NHS), 1‐ethyl‐3(3‐dimethylaminopropyl)‐carbodiimide (EDC), bovine serum albumin (BSA) and hydrogen tetrachloroaurate (III) trihydrate (HAuCl_4_·3H_2_O), tri(2‐carboxyethyl) phosphine hydrochloride (TCEP), were purchased from Sigma‐Aldrich (USA). Potassium ferricyanide (K_3_[Fe(CN)_6_]) and potassium ferrocyanide (K_4_[Fe(CN)_6_]) were purchased from Shanghai Chemical Reagent Co. Ltd. (Shanghai, China). Rabbit polyclonal anti‐cTnI antibody, mouse monoclonal anti‐cTnI antibody were purchased from Proteintech (Shanghai, China). cTnI recombinant protein was obtained from Abcam company (USA). Tris(2,2‐bipyridyl) dichlororuthenium (II) hexahydrate (Ru(bpy)_3_Cl_2_·6H_2_O), cyclohexane, 1‐hexanol, tetraethyl orthosilicate (TEOS), NaCl, trehalose, triton X‐100, polyvinylpyrrolidone (PVP), tween 20, tripropylamine (TPrA), ammonium hydroxide (NH_3_·H_2_O), and ethanol were all from Aladdin Chemistry Co., Ltd. (Shanghai, China). Human serum matrix was purchased from Randox (UK). Artificial saliva (containing water, mucin and amylase), urine (containing calcium chloride, magnesium chloride, sodium chloride, potassium chloride, phosphate, ammonium chloride, and urea) and interstitial fluid samples (containing glucose, salt, fatty acids, and minerals) were bought from Biochemazone Co., Ltd. (Canada). Fetal bovine serum was purchased from Gibco Life Technologies. All saliva, urine and interstitial fluid samples involving animals were obtained from Shanghai Yanjin Biological. Antibody concentration was determined using the enhanced BCA protein assay kit from Labgic Co., Ltd. (Beijing, China). ELISA kit for detection of cTnI was bought from Beijing Solarbio Technology Co., Ltd.

Electrochemical tests consisted of a 1.8 mm diameter carbon electrode as the working electrode, Ag/AgCl as the reference electrode, and 1.8 mm diameter carbon electrode as the counter electrode to form a three‐electrode system. The electrochemical workstation was purchased from Wuhan Meoguan Biotechnology Co., Ltd. The H10682‐210 photomultiplier tube (PMT) and the CH297‐011 photon counting unit were purchased from Hamamatsu Photonics Co., Ltd. AC voltage was provided by a SDG‐1062X function/arbitrary waveform generator (SIGLENT) and observed by a DSOX3014T oscilloscope (Keysight Technologies). Electrochemical impedance spectroscopy (EIS) test results were recorded by a ZAHNER ZENNIUM CIMPS‐1 electrochemical workstation.

The morphology of the samples was observed under a HT7700 exalens transmission electron microscope (TEM) (Japan) at an accelerating voltage of 120 kV. UV‐vis was carried out via a PE‐Lambda 35 (PerkinElmer, United States). Zetasizer Nano (Malvern) was used to test the potential and the size of different nanoparticles with different modifications in solution. X‐ray photoelectron spectroscopy (XPS) data was recorded by a PHI 5000C&PHI5300 electron spectrometer (PHI, United States). The capillary (length of 80 mm, diameter of 1.4 mm) was used to monitor the water phase separation states. The photographs of the capillary channel were recorded by optical microscope (eyepiece × 10, objective lens × 4) from SuZhou ShenYing Optical Co., Ltd.

### Synthesis of Ru@SiO_2_ NPs

Ru@SiO_2_ NPs was synthesized following the previous reports with minor modifications.^[26b]^ First, 7.5 mL of cyclohexane, 1.77 mL of Triton X‐100, 1.6 mL of n‐hexanol, 400 µL of distilled water, and 400 µL of 40 mM Ru(bpy)_3_^2+^ were mixed under vigorous stirring for 30 min. Then, 100 µL of distilled water and 60 µL of NH_3_·H_2_O were added into the homogeneous solution and kept stirring for 24 h. After that, 10 mL of acetone was added to obtain the orange nanoparticles. The obtained nanoparticles were centrifuged and re‐dispersed in 6 mL of ethanol and 200 µL of APTES. The obtained products were washed with ethanol and water three times repeatedly. Finally, the particles were collected and dried at 37 °C.

### Synthesis of AuNPs

AuNPs were synthesized based on the previous work.^[^ ^22^ ^]^

### Preparation of Ru@SiO_2_ NPs‐Ab_2_

Ru@SiO_2_ NPs‐Ab_2_ conjugates were synthesized via EDC/NHS coupling. First, Ab_2_ was activated by adding 100 µL of EDC/NHS (400 mM/100 mM) for 1 h at room temperature.^[^ ^22^ ^]^ Then 5 mg of Ru@SiO_2_ NPs were dispersed in 990 µL of PBS and ultrasonicated homogeneously and mixed with activated antibody. After 2 h of incubation with 600 rpm agitation, the obtained solution was blocked with 20 µL of PBS containing 1 M glycine for 30 min and 100 µL of PBS containing 10 wt.% BSA for another 30 min. Later, the mixture was centrifuged to remove excess reagents and re‐dispersed in 500 µL of PBS containing 0.1 wt.% BSA.

### Preparation of Ru(bpy)_3_^2+^‐Probe DNA

Ru(bpy)_3_^2+^‐probe DNA was prepared following the previous report. First, 1 mL of carboxyl Ru(bpy) _3_^2+^ was activated by 100 µL of EDC/NHS (400 mM/100 mM) and incubated for 30 min at room temperature. Then 100 µL of 10 µM probe DNA was added and kept stirring for 2 h. Then the obtained solution was filtered through a dialysis membrane (1 kDa) for 4 h and stored at 4 °C for further use.

### AC‐Driven ECL‐M POCT Device Measurement

The electrode layer was cleaned in ethanol and dried at room temperature. 2.5 µL of blocking solution involving 10 mg mL^−1^ polyvinylpyrrolidone, 10 mg mL^−1^ BSA, 50 mg mL^−1^ trehalose, and 0.1% Tween 20 were dipped on the lower cover at 37 °C for 8 h to promote the release efficiency of Ru@SiO_2_ NPs‐Ab_2_ and Ru(bpy)_3_^2+^/probe DNA conjugates. For detection of cTnI, 2.5 µL of Ru@SiO_2_ NPs‐Ab_2_ conjugates were covered over the blocking sites. 2 µL of AuNPs were dropped on working electrode 1 (WE_1_) until dried in the air. 1.5 µL of 250 µg mL^−1^ Ab_1_ was immobilized on the AuNPs‐coated WE_1_ via Au‐N covalent bond at 4 °C for a whole night. Before the assembly of the ECL‐M chip, the modified WE_1_ was washed with distilled water. cTnI stock antigen was serially diluted using 100% human serum matrix, and the final assay sample solution was diluted ten‐fold of the prepared cTnI antigen solution using 1% BSA, 3% NaCl, 0.2% Triton X‐100 (TX‐100) in PBS (pH 7.4) to produce a 10% concentration of human serum matrix in each sample. The AC‐voltage was applied at an amplitude of 9 V_pp_ (peak‐to‐peak voltage) and a frequency of 1 kHz. 5 min after adding 35 µL of the sample, 5 µL of the engineered fluid containing 1% TX‐100 was injected. For detection of miR‐499‐5p, 2.5 µL of Ru(bpy)_3_^2+^/probe DNA conjugates were coated on the blocking sites. First, 2 µL of AuNPs were dropped on working electrode 2 (WE_2_) until dried in the air. 1.5 µL of 100 µM capture DNA was previously activated by 10 mM TCEP and then incubated on the WE_2_ at 4 °C for a whole night. miR‐499‐5p standard solution was serially diluted using DEPC water, and the final assay solution was diluted ten‐fold of the prepared miR‐499 solution using 1% BSA, 3% NaCl, 0.2% TX‐100 in PBS (pH 7.4). Then ECL measurement was initiated by adding 50 µL of ECL reaction PBS (pH 7.4) buffer containing 0.1 M TPrA and 1% TX‐100 as ECL co‐reactant. To detect ECL signals, cyclic voltammetry was utilized with the scan voltage from 0 to 2.0 V at the scan rate of 100 mV s^−1^.

### Rat Model of AMI

Animals were treated under the Guidelines for the Care and Use of Laboratory Animals of Fudan University. All animal experimental protocols were approved by the Animal Ethics Committee of Fudan University, China (2021JSCHEM‐020). 16 rats were divided evenly into two groups, including AMI model group (8 rats) and control group (8 rats). The rats in the control group were free to access water and food, while rats in AMI model group were fasted for 12 h before operation. Rats of AMI model group were established by Shanghai Yanjin Biological. Ltd via ligation of the left anterior descending coronary artery of rat. Then, saliva, urine, and interstitial fluid samples of all rats were collected every 0 h, 1 h, 2 h, 4 h, 8 h, 16 h, 24 h, 32 h, and 48 h post AMI and stored at −80 °C for further analysis.

## Conflict of Interest

The authors declare no conflict of interest.

## Author Contributions

All authors have given approval to the final version of the manuscript. Huiwen Xiong, Chenxin Zhu and Xueen Fang conceived the idea of the ECL‐M devices. Huiwen Xiong, Chenxin Zhu, Pintao Li and Xueen Fang designed the ECL microfluidic chip. Huiwen Xiong and Chenxin Zhu conducted experiments of optimization, characterization, performance evaluation, sample testing and data analysis. Changhao Dai and Pintao Li conducted partial experiments of sample testing. Xin Ye, Yuanyuan Li and Huali Shen provided clinical samples. Shuang Yang analyzed data of sample testing experiments. Ghazala Ashraf revised the manuscript. Huiwen Xiong, Chenxin Zhu, Changhao Dai, Pintao Li and Xueen Fang wrote the manuscript. Dacheng Wei, Hui Chen, Huali Shen, Jilie Kong and Xueen Fang supervised the study.

## Supporting information

Supporting Information

Supplemental Movie 1

Supplemental Table 1

## Data Availability

The data that support the findings of this study are available from the corresponding author upon reasonable request.
