# Supplementary material for: An Alternating Current Electroosmotic Flow‐Based Ultrasensitive Electrochemiluminescence Microfluidic System for Ultrafast Monitoring, Detection of Proteins/miRNAs in Unprocessed Samples
Source: Adv Sci (Weinh). 2023 Dec 9;11(6):2307840. doi: 10.1002/advs.202307840 (PMC10853704; doi:10.1002/advs.202307840)
Supplement: Supplementary file 1 — Supporting Information [file ADVS-11-2307840-s002.pdf]

ADVANCED SCIENCE
Open Access

Supporting Information

for *Adv. Sci.*, DOI 10.1002/advs.202307840

An Alternating Current Electroosmotic Flow-Based Ultrasensitive Electrochemiluminescence Microfluidic System for Ultrafast Monitoring, Detection of Proteins/miRNAs in Unprocessed Samples

Huiwen Xiong, Chenxin Zhu, Changhao Dai, Xin Ye, Yuanyuan Li, Pintao Li, Shuang Yang, Ghazala Ashraf, Dacheng Wei, Hui Chen, Huali Shen*, Jilie Kong* and Xueen Fang**

Supporting information

An alternating current electroosmotic flow-based ultrasensitive electrochemiluminescence microfluidic system for ultrafast monitoring, detection of proteins/miRNAs in unprocessed samples

Huiwen Xiong [a, #], Chenxin Zhu [b, #], Changhao Dai [c], Xin Ye [d], Yuanyuan Li [e], Pintao Li [a], Shuang Yang [b], Ghazala Ashraf [a], Dacheng Wei [c], Hui Chen [a, *], Huali Shen [b, *], Jilie Kong [a, *], and Xueen Fang [a, *]

[a] Department of Chemistry, Fudan University, Shanghai 200438, China
[b] Institutes of Biomedical Sciences and Minhang Hospital, Fudan University, Shanghai 200032, China.
[c] State Key Laboratory of Molecular Engineering of Polymers, Department of Macromolecular Science, Fudan University, Shanghai 200438, China
[d] Department of Laboratory Medicine, the First Affiliated Hospital of Xi'an Jiaotong University, Xi'an 710061, Shaanxi, PR China
[e] Yizheng Hospital of TCM, Yangzhou 211400, China
Corresponding Author
*Hui Chen, chenhui@fudan.edu.cn;
*Huali Shen, shenhuali@fudan.edu.cn;
*Jilie Kong, jlkong@fudan.edu.cn.
*Xueen Fang, fxech@fudan.edu.cn;
Co-first Author
[#] Huiwen Xiong and Chenxin Zhu contributed equally to this work.
Author Contributions
All authors have given approval to the final version of the manuscript.

Contents

Table S1 The sequences (5'-3') of miR-499-5p, capture and probe DNA in this work.
Table S2 Summary of cost prices for cTnI test kit.
Table S3 The cost of the components of ECL-M POCT device.
Table S4 Summary of cost prices for chemiluminescence detection systems.
Table S5 Parameters of COMSOL simulation.
Table S6 Molecular interaction between cTnI antibody and cTnI antigen.
Table S7 Sequences of other miRNA interferences.
Table S8 Clinical information on patient samples.
Table S9 Comparison of different methods for detection of cTnI in AMI diagnosis.
Table S10 Comparison of different methods for amplification-free detection of miRNA in clinical diagnosis.
Figure S1 Size of the designed ECL-M POCT device. Noteworthily, the height of the enclosed device was 135 mm and was 265 mm after opening the cover.
Figure S2 (a) Overall dimension diagram of microfluidic chip. (b) Schematic diagram of position and size of flow channel and electrodes.
Figure S3 (a) Front and (b) back photograph of the ECL-M chip.
Figure S4 AC voltage with a form of square wave was applied to the ECL-M chip which produced an alternating electric field between the two Ag electrodes.
Figure S5 COMSOL simulation of electrical field under the voltage of -4.5 V (a) and +4.5 V (b).
Figure S6 TEM images of AuNPs (a) and Ru@SiO_2 NPs (b). Scale bars, 50 nm (a) and 50 nm (b).
Figure S7 Statistical analyses of the size distributions of AuNPs (a) and Ru@SiO_2 NPs (b).
Figure S8 UV-vis adsorption spectra of AuNPs, $Ru(bpy)_3^{2+}$, Ru@SiO_2 NPs.
Figure S9 Zeta potential of $Ru(bpy)_3^{2+}$, Ru@SiO_2 NPs, Ru@SiO_2-NH_2 and AuNPs.
Figure S10 AFM image (in 1× TM buffer) of Au electrode (a) and antibody immobilized on Au electrode (b) at 4 °C for one night. The color bar indicates the height of the scanned surface.
Figure S11 BCA analysis for Ab_1 concentration in the ECL-M system.
Figure S12 XPS spectroscopy of N 1s peak on AuNPs modified silicon wafer and Ab_1 immobilized on AuNPs modified silicon wafer.
Figure S13 Micrograph of dyed PBS separated by the engineered fluid containing 1% TX-100 in the capillary. The first injection was dyed yellow with potassium ferricyanide. The ECL reaction solution was dyed blue with methylene blue.
Figure S14 ECL measurement of blank versus cTnI (concentration of 10 pg/mL) under three different conditions: without electric field, with DC-driven field and with AC-driven field. The working solution was PBS (pH 7.4) containing 0.1 M TPrA. Scan rate was 100 mV/s.
Figure S15 COMSOL simulation model and boundary of ECL-M channel.
Figure S16 Prediction of antigen-antibody interaction of cTnI. The X-ray crystal structures of a chicken anti-cardiac Troponin I scFv (4P48) and cardiac troponin C-

troponin I complex (1MXL) were obtained from the Protein Data Bank. Multiple groups of residues were used to form hydrogen bonds between anti-cTnI antibody (purple one) and cTnI antigen (blue one), such as the hydrogen bond formed by Ser-199 of anti-cTnI and Lys-17 of cTnI.

Figure S17 Photograph of the sample tested solution viscosity.

Figure S18 Temperature field of the ECL-M channel under ACET effect.

Figure S19 Velocity distribution of ACET-induced Antigen solution in ECL-M channels.

Figure S20 Photograph of the electrical conductivity of antigen solution.

Figure S21 Velocity distribution of ACEO-induced Antigen solution in ECL-M channels.

Figure S22 EIS characterization of the ECL-M POCT biosensor corresponding with each modification step. a-h, CE (a), AuNPs/CE (b), Ab_1/AuNPs/CE (c), cTnI/Ab_1/AuNPs/CE (d), Ru@SiO_2-Ab_2/cTnI/Ab_1/AuNPs/CE (e), capture DNA/AuNPs/CE (f), miR-499/capture DNA/AuNPs/CE (g), $Ru(bpy)_3^{2+}$-probe DNA/miR-499/capture DNA/AuNPs/CE (h). The concentration of cTnI was 1 pg/mL and miR-499-5p was 100 aM. The EIS working solution was 5 mM $[Fe(CN)_6]^{3-/4-}$ solution containing 0.1 M KCl. Scan rate was 100 mV/s.

Figure S23 Synchronized electrochemical measurement of cTnI with (red curve) and without (blue curve) the AC voltage driven force under the voltage of 0~+2.0 V. The concentration of cTnI was 1 pg/mL. The working solution was PBS (pH 7.4) containing 0.1 M TPrA. Scan rate was 100 mV/s.

Figure S24 Optimization of experimental conditions. Optimization of V_{pp} of AC (a), AC-driven incubation time of cTnI (b), AC-driven incubation time of miR-499-5p (c).

Figure S25 Optimization of experimental conditions. a, b, c, Optimization of concentration of capture DNA (a), Ab_1 (b), AuNPs (c).

Figure S26 cTnI and miR-499-5p detection in different biological sample solutions (PBS, artificial saliva, urine, interstitial fluid, and serum). Significant difference between ECL signal of PBS and other biological samples was determined by one-way ANOVA followed by a t test. *$p < 0.05$, **$p < 0.01$, ***$p < 0.001$, ****$p < 0.0001$ vs. Blank; n.s., no significant.

Figure S27 Time-course Infarct photograph of rat models. Once the blood collection was finished, rats were put to death after anesthetized with pentobarbital. Hearts were rapidly removed and sliced transversely into five sections. The hearts were soaked in 10% buffered formalin and frozen in -20 °C for 20 minutes. Five slices were incubated with 1% triphenyl tetrazolium chloride (TTC) at 37°C for 15 minutes under dark conditions to observe the infarction area. The normal heart tissues were stained with red color while infarct heart areas were white.

Figure S28 (a) Comparison of ECL-M POCT device (this work) with detection of cTnI with other methods. Incubation time and LOD were estimated and compared with other methods. (b) Comparison of ECL-M POCT device (this work) with detection of miR-499-5p by other methods. Incubation time, reaction temperature, and LOD were estimated and compared with other methods.

Table S1 The sequences (5'-3') of miR-499-5p, capture and probe DNA in this work.

Name	Sequence (5'-3')
miR-499-5p	UUA AGA CUU GCA GUG AUG UUU
Capture DNA	SH-C6-TAC GCC ACT AGC TCC AAA CAT CAC
Probe DNA	TGC AAG TCT TAA-C6-NH_2

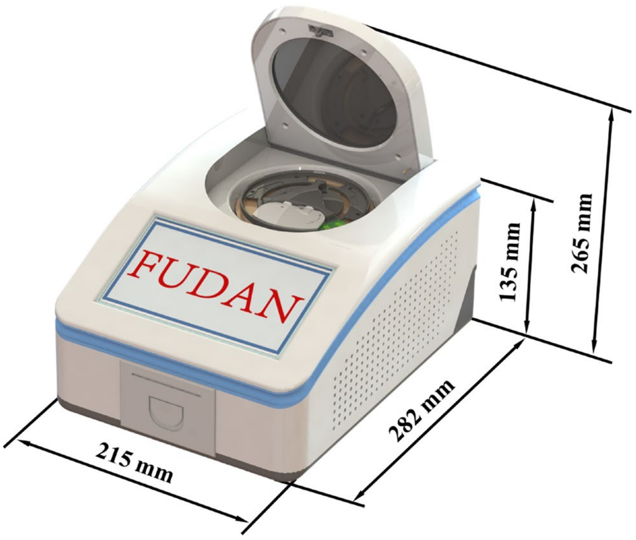

Figure S1 Size of the designed ECL-M POCT device. Noteworthily, the height of the enclosed device was 135 mm and was 265 mm after opening the cover.

Table S2 Summary of Cost Prices for cTnI test kit.

Test kit	Supplier	Price (USD)
ECL-M	/	0.15
Chemiluminescence	SUN biotech	2.05
Chemiluminescence	Tailored Medical	2.22
Magnetic particle chemiluminescence immunoassay	Bioscience	1.37
Magnetic particle chemiluminescence immunoassay	Perfebio	1.71
Magnetic particle chemiluminescence immunoassay	Biochip Langdao	4.5

***The price in the table is for one-person test.**

Table S3 The cost of the components of ECL-M POCT device.

**Components**	**Supplier**	**Cost (USD)**
PMT	Hamamatsu Photonics	1528
Photon counting unit	Hamamatsu Photonics	170
The electrochemical workstation	Wuhan Meoguan Biotechnology	420
Control unit	/	300
Rotation mechanism	Oriental motor	165
Structure platform	/	500
Others	/	300
Total cost	/	3383

Table S4 Summary of Cost Prices for Chemiluminescence Detection Systems.

**Device**	**Supplier**	**Price (USD)**
ECL-M system	/	3383
Shine i1900	Increcare	4980
MAGLUMI X3	Snibe Diagnostic	15000
CL-900i	Mindray	22000

***The equipment prices in the table are cost prices.**

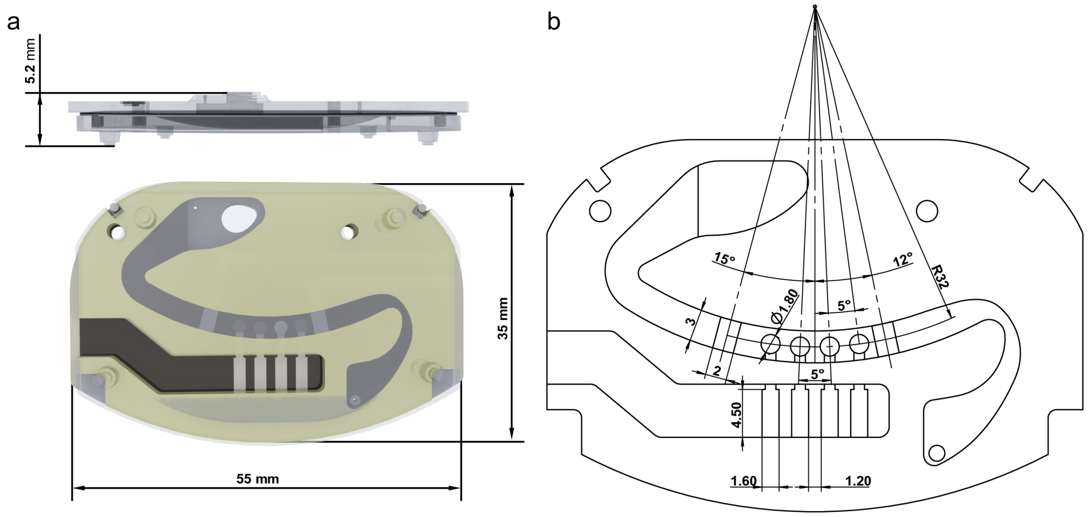

Figure S2 (a) Overall dimension diagram of microfluidic chip. (b) Schematic diagram of position and size of flow channel and electrodes.

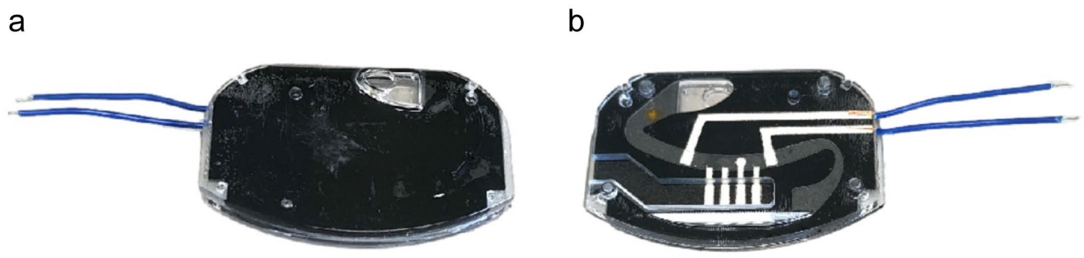

Figure S3 (a) Front and (b) back photograph of the ECL-M chip.

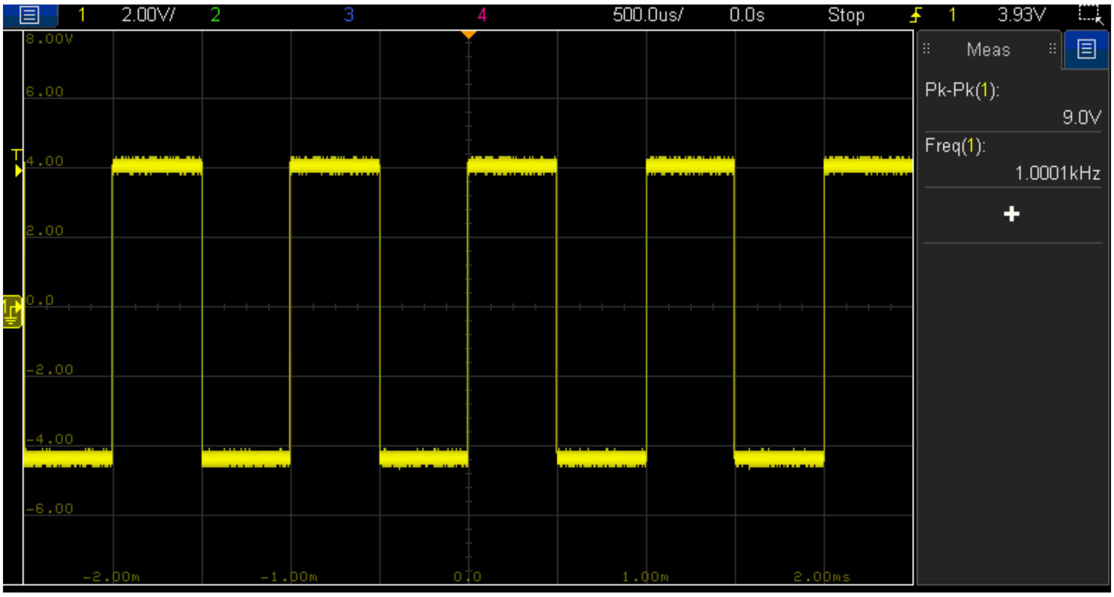

Figure S4 AC voltage with a form of square wave was applied to the ECL-M chip which produced an alternating electric field between the two Ag electrodes.

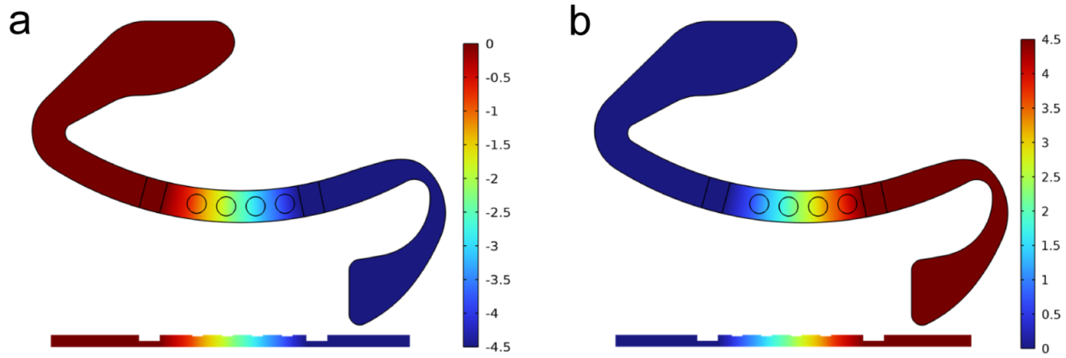

Figure S5 COMSOL simulation of electrical field under the voltage of -4.5 V (a) and +4.5 V (b).

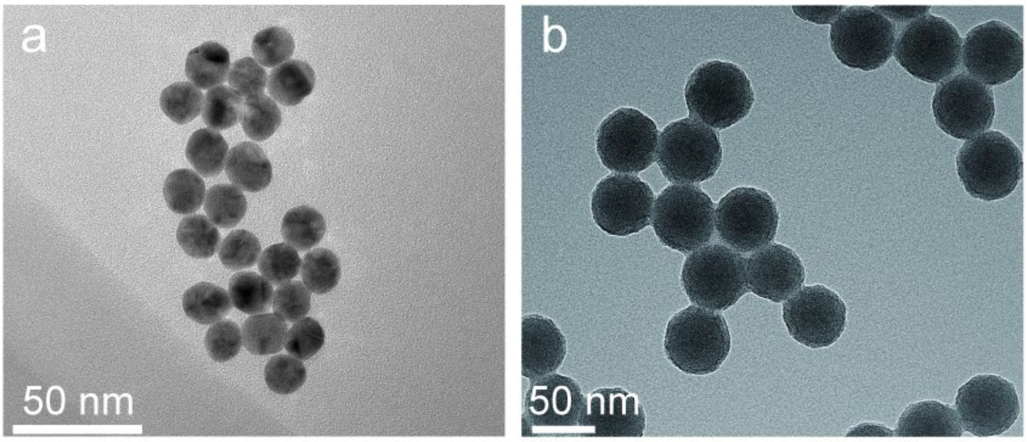

Figure S6 TEM images of AuNPs (a) and Ru@SiO_2 NPs (b). Scale bars, 50 nm (a) and 50 nm (b).

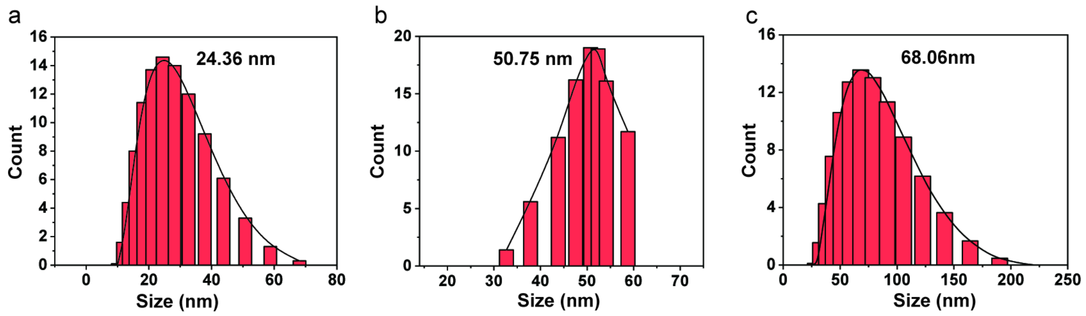

Figure S7 Statistical analyses of the size distributions of AuNPs (a) and $Ru@SiO_2$ NPs (b), $Ru@SiO_2$ NPs-Ab_1 (c).

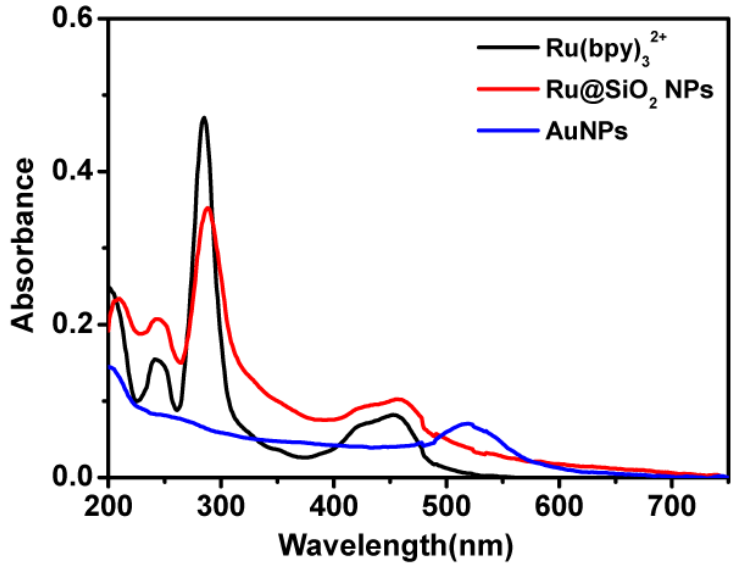

Figure S8 UV-vis adsorption spectra of AuNPs, $Ru(bpy)_3^{2+}$, Ru@SiO_2 NPs.

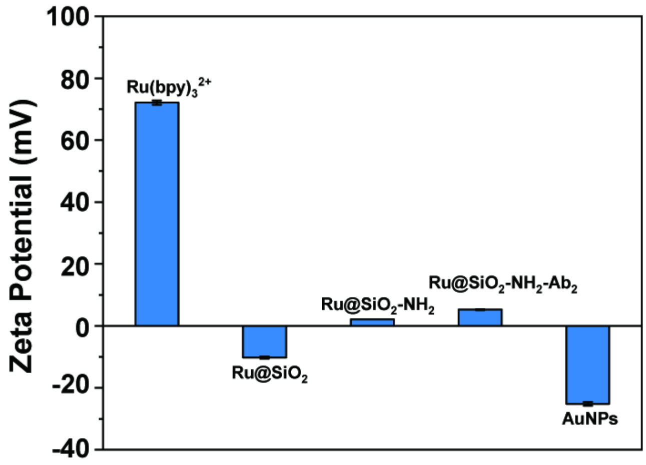

Figure S9 Zeta potential of $Ru(bpy)_3^{2+}$, $Ru@SiO_2$ NPs, $Ru@SiO_2$-NH_2, $Ru@SiO_2$ NPs-Ab_1 and AuNPs.

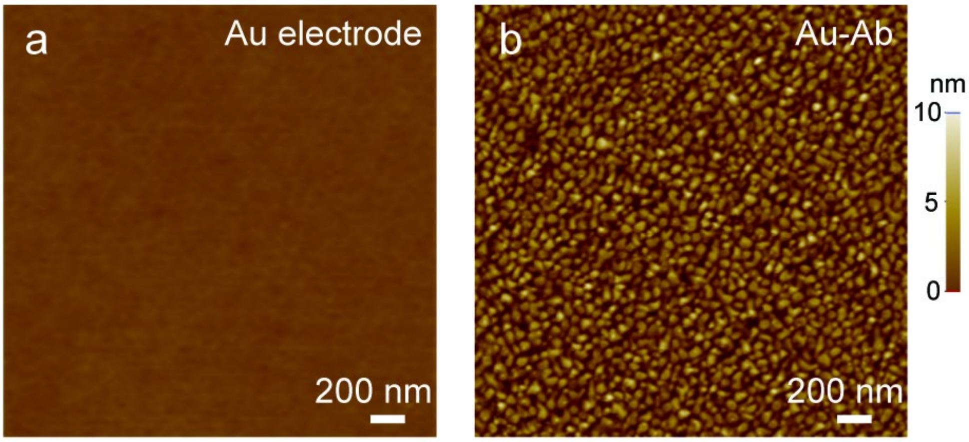

Figure S10 AFM image (in 1× TM buffer) of Au electrode (a) and antibody immobilized on Au electrode (b) at 4 ℃ for one night. The color bar indicates the height of the scanned surface.

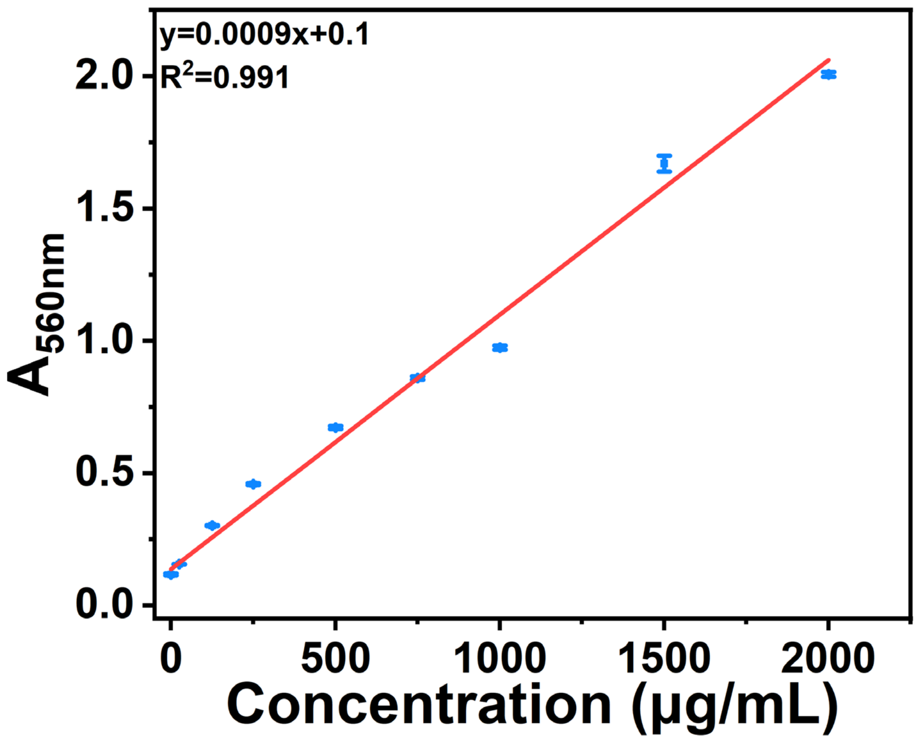

Figure S11 BCA analysis for Ab_1 concentration in the ECL-M system.

To explain the load efficiency of Ab_1 loading on AuNPs, 20 μL of AuNPs were mixed with 15 μL of 250 μg/mL Ab_1 at 4 °C for a whole night. The mixture was then centrifuged at 12000 rpm for 10 min, followed by redispersed in 15 μL PBS solution (pH 7.4). Then the mixture solution was analyzed following the quantification of the protein concentration by the BCA method (Figure S11). The final concentration of Ab_1 was estimated as 79.2 μg/mL, showing the effective loading efficiency of 31.7 % in the protein analysis system.

To explain the load efficiency of capture DNA loading on AuNPs, we tested the concentration of capture DNA by a K5600 ultra-micro spectrophotometer.

20 μL of AuNPs were mixed with 15 μL of 100 μM capture DNA at 4 °C for a whole night. The mixture was then centrifuged at 12000 rpm for 10 min, followed by redispersed in 15 μL PBS solution (pH 7.4). Then the mixture solution was analyzed following the quantification of the protein concentration by the BCA method (Figure S11). The final concentration of capture DNA was estimated as 92.4 μM, showing the effective loading efficiency of 92.4 % in the protein analysis system.

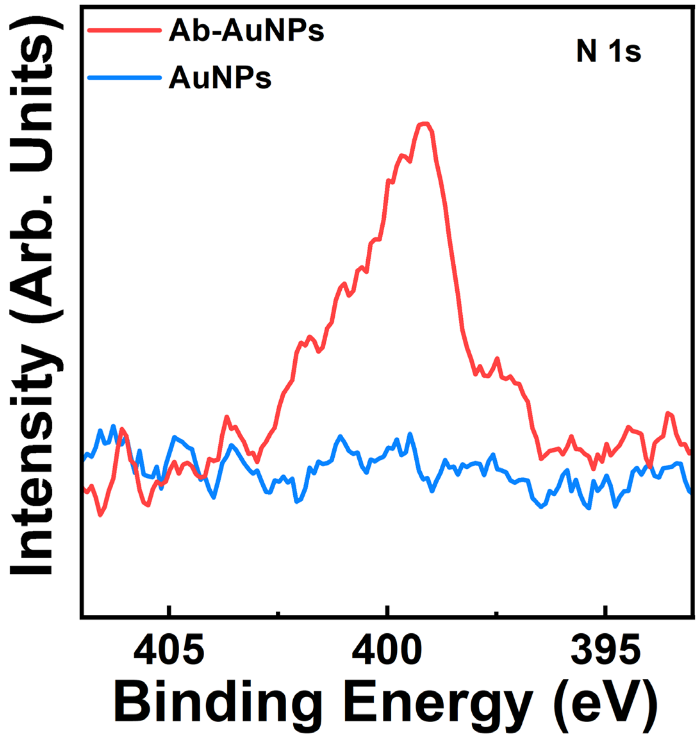

Figure S12 XPS spectroscopy of N 1s peak on AuNPs modified silicon wafer and Ab_1 immobilized on AuNPs modified silicon wafer.

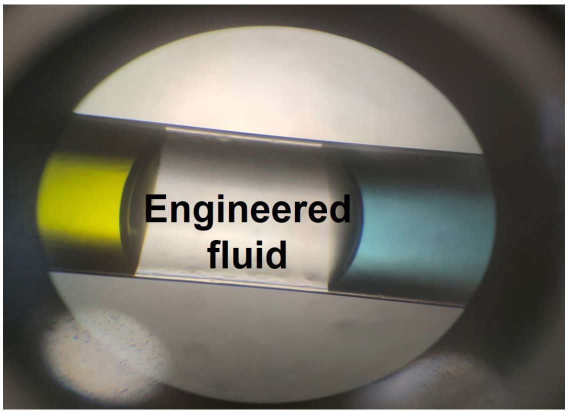

Figure S13 Micrograph of dyed PBS separated by the engineered fluid containing 1% TX-100 in the capillary. The first injection was dyed yellow with potassium ferricyanide. The ECL reaction solution was dyed blue with methylene blue.

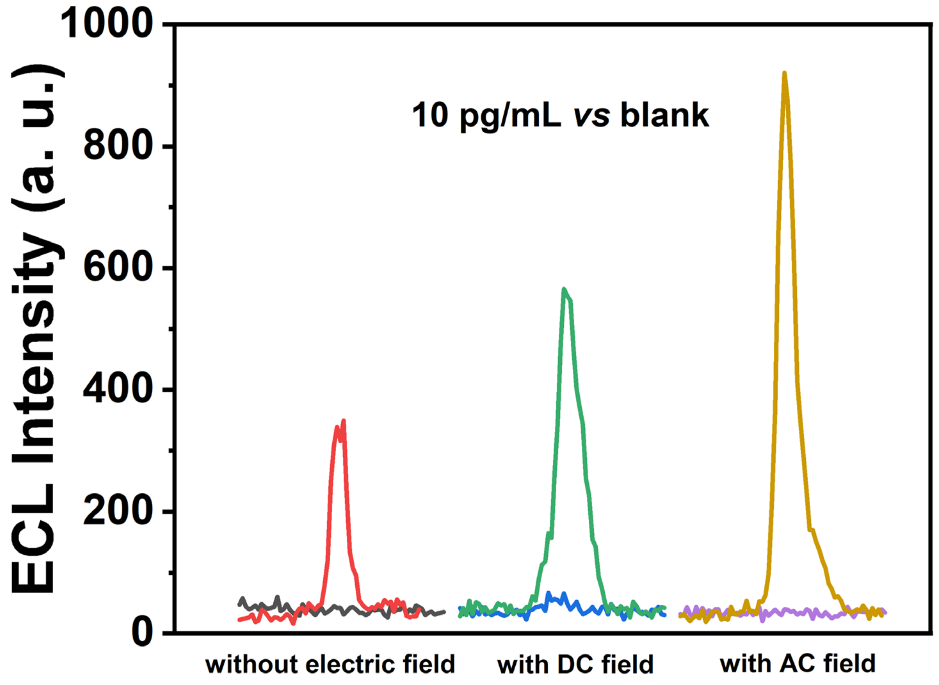

Figure S14 ECL measurement of blank versus cTnI (concentration of 10 pg/mL) under three different conditions: without electric field, with DC-driven field and with AC-driven field. The working solution was PBS (pH 7.4) containing 0.1 M TPrA. Scan rate was 100 mV/s.

Table S5 Parameters of COMSOL simulation.

**Parameter**	**Value**	**Unit**
Viscosity	0.00153	Pa*s
Density	1000	kg/m^3
Relative dielectric constant	79	/
Diffusion coefficient	$2209*10^{-6}$	mm^2/s
Temperature	298	K
Constant pressure heat capacity	4200	J/(kg*K)
Thermal conductivity	0.6095	W/(m*K)
Initial antibody concentration	250	μg/mL
Antigen initial concentration	10	pg/mL

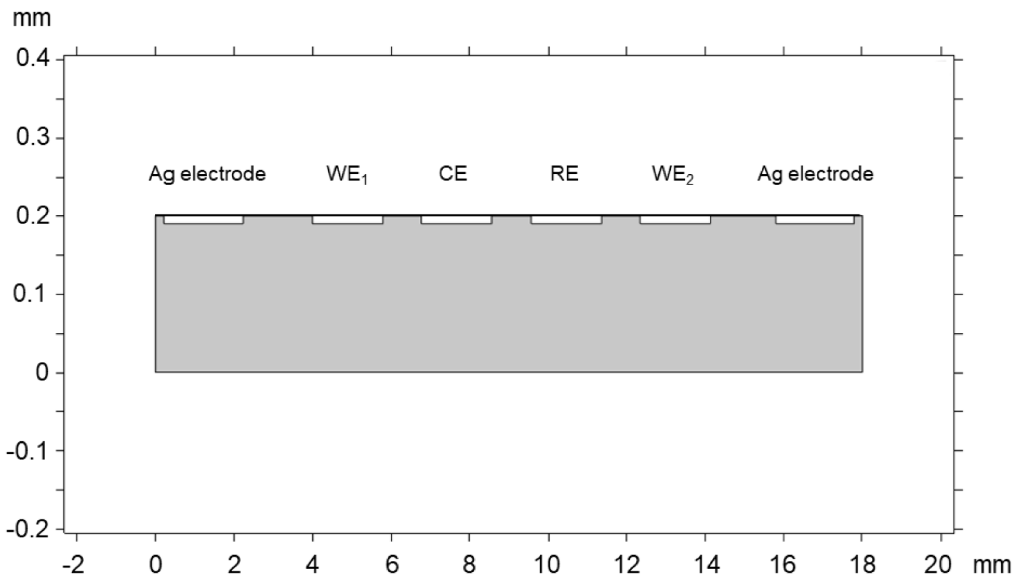

Figure S15 COMSOL simulation model and boundary of ECL-M channel.

Table S6 Molecular interaction between cTnI antibody and cTnI antigen.

Receptor	Ligand	Hydrogen bond interaction (2.0Å)[a]	Electrostatic interaction
cTnI antibody (4P48)	cTnI antigen (1MXL)	A:199:Ser-B:17:Lys<br>A:201:Asp-B:18:Asn<br>A:208:Arg-B:9:Val<br>A:212:Asn-B:10:Glu	\

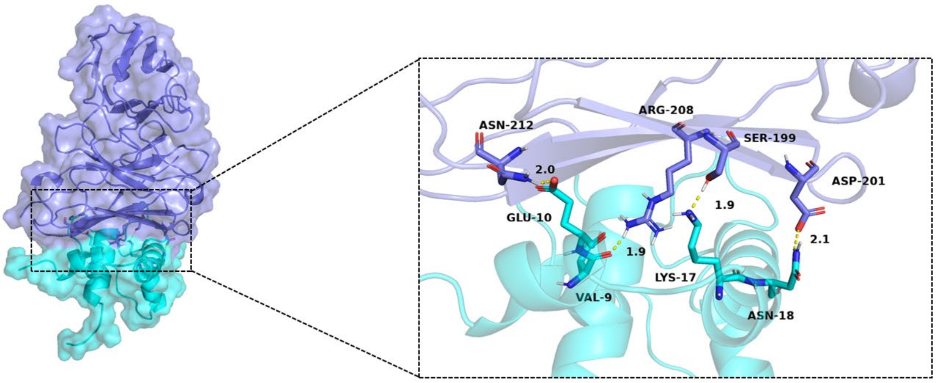

Figure S16 Prediction of antigen-antibody interaction of cTnI. The X-ray crystal structures of a chicken anti-cardiac Troponin I scFv (4P48) and cardiac troponin C-troponin I complex (1MXL) were obtained from the Protein Data Bank. Multiple groups of residues were used to form hydrogen bonds between anti-cTnI antibody (purple one) and cTnI antigen (blue one), such as the hydrogen bond formed by Ser-199 of anti-cTnI and Lys-17 of cTnI.

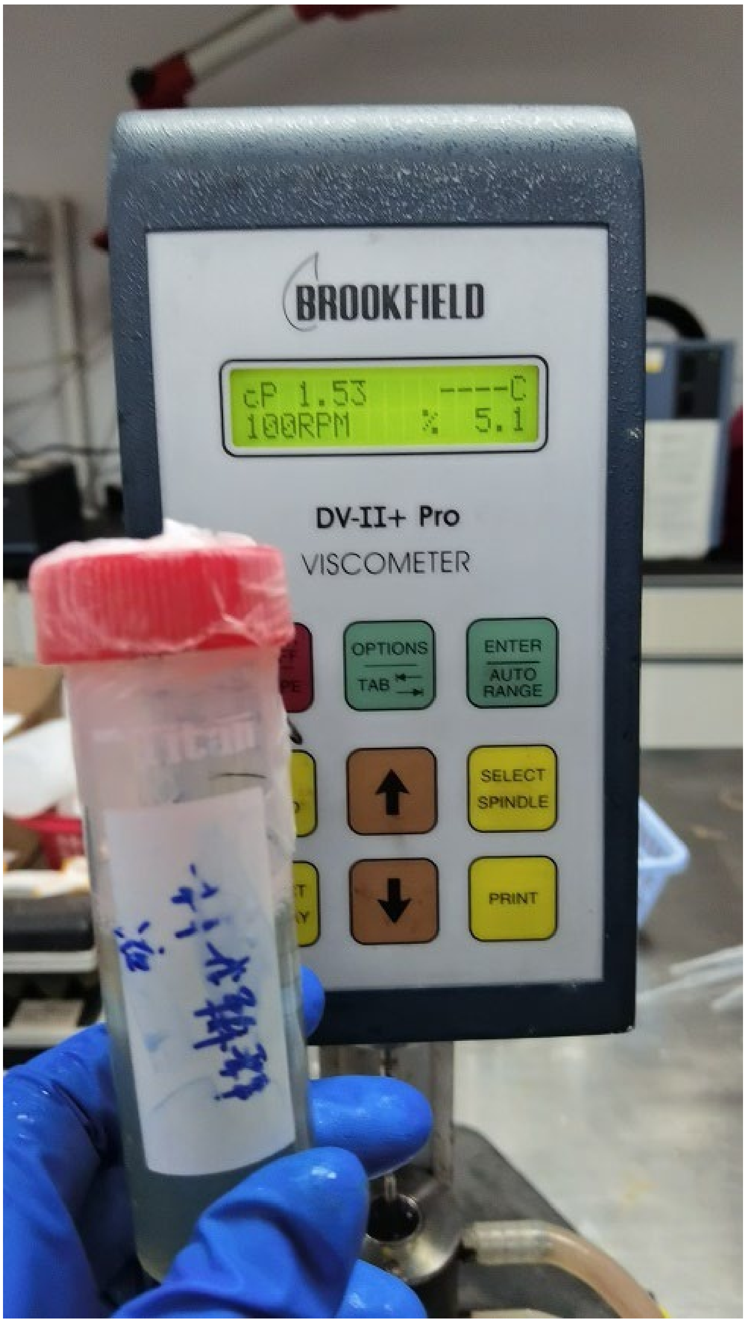

Figure S17 Photograph of the sample tested solution viscosity.

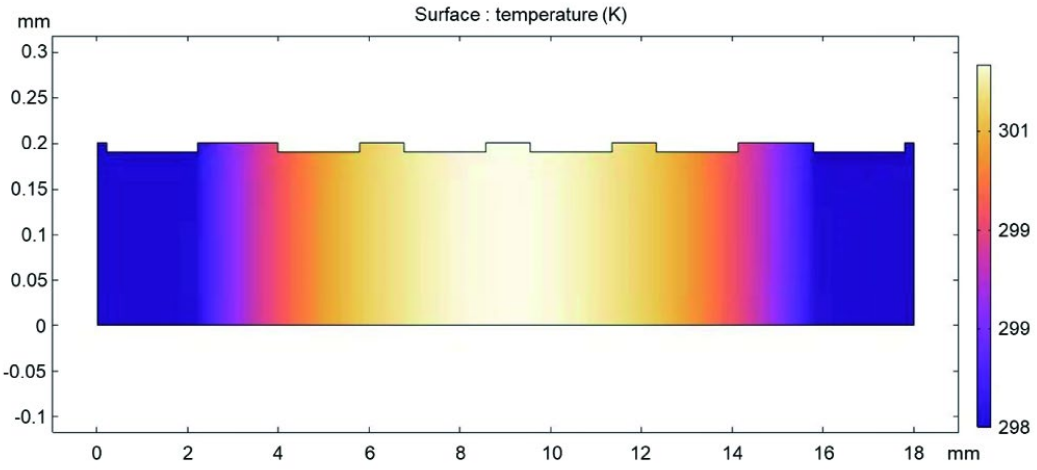

Figure S18 Temperature field of the ECL-M channel under ACET effect.

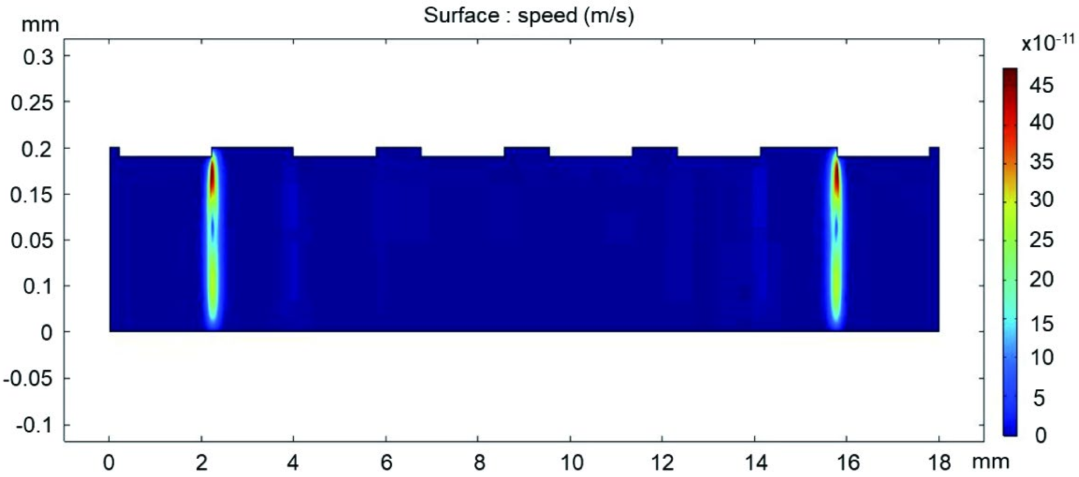

Figure S19 Velocity distribution of ACET-induced Antigen solution in ECL-M channels.

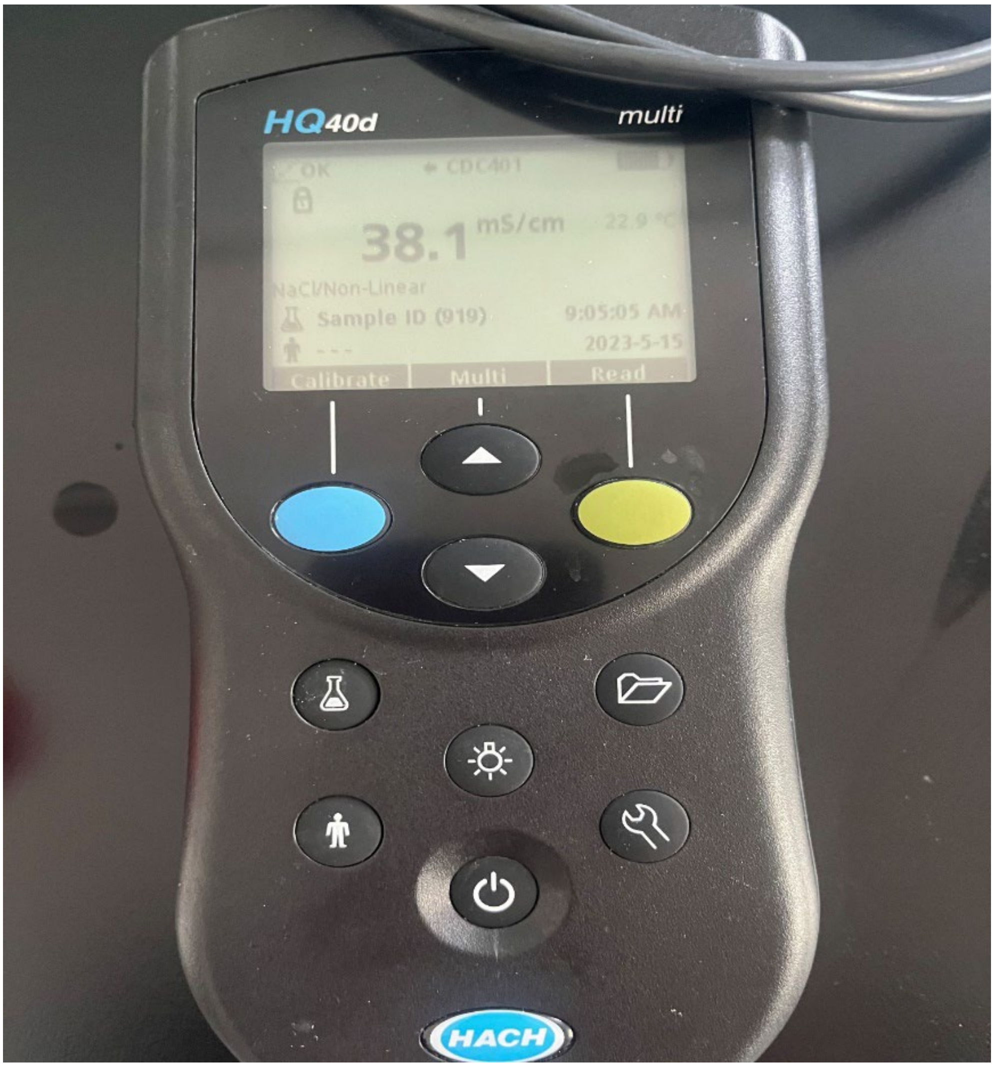

Figure S20 Photograph of the electrical conductivity of antigen solution.

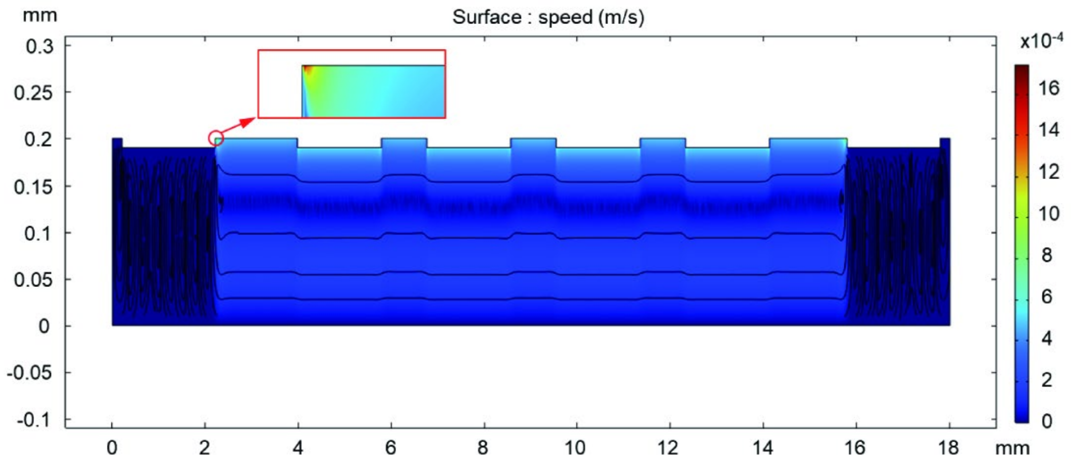

Figure S21 Velocity distribution of ACEO-induced Antigen solution in ECL-M channels.

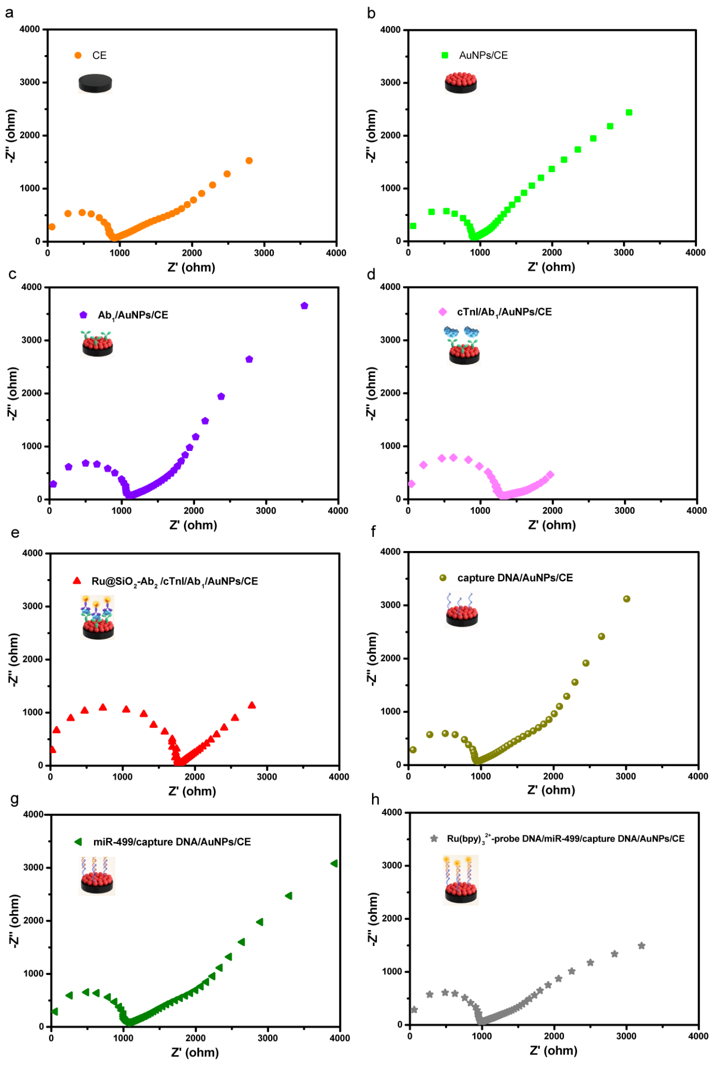

Figure S22 EIS characterization of the ECL-M POCT biosensor corresponding with each modification step. a-h, CE (a), AuNPs/CE (b), Ab_1/AuNPs/CE (c), cTnI/Ab_1/AuNPs/CE (d), Ru@SiO_2-Ab_2/cTnI/Ab_1/AuNPs/CE (e), capture DNA/AuNPs/CE (f), miR-499/capture DNA/AuNPs/CE (g), $Ru(bpy)_3^{2+}$-probe DNA/miR-499/capture DNA/AuNPs/CE (h). The concentration of cTnI was 1 pg/mL and miR-499-5p was 100 aM. The EIS working solution was 5 mM $[Fe(CN)_6]^{3-/4-}$ solution containing 0.1 M KCl. Scan rate was 100 mV/s.

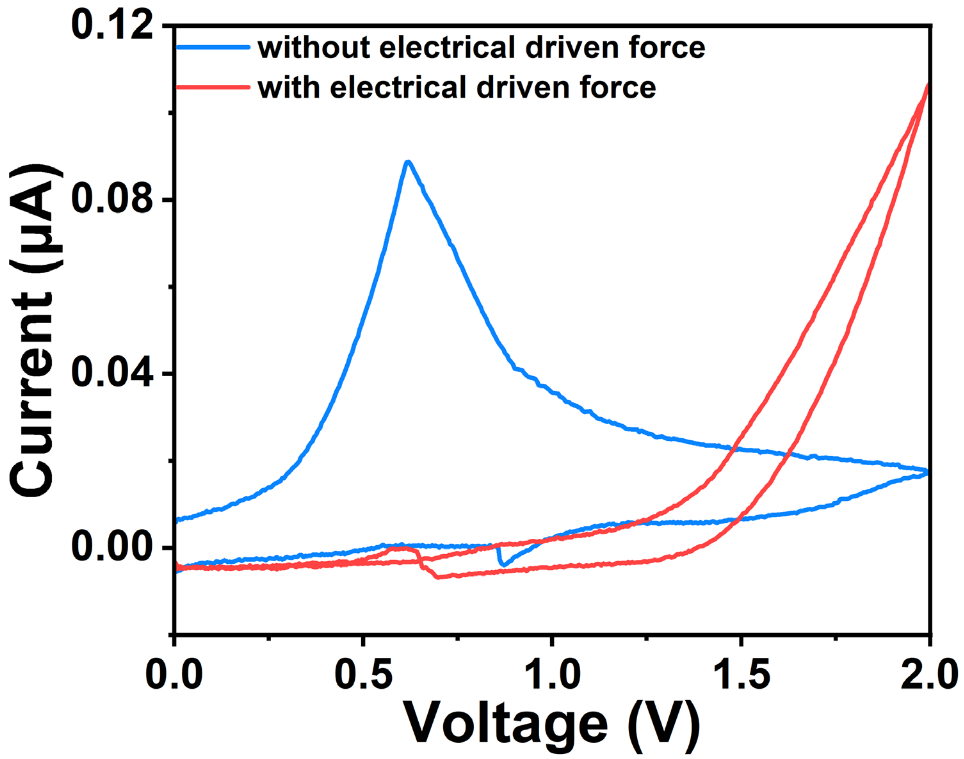

Figure S23 Synchronized electrochemical measurement of cTnI with (red curve) and without (blue curve) the AC voltage driven force under the voltage of 0~+2.0 V. The concentration of cTnI was 1 pg/mL. The working solution was PBS (pH 7.4) containing 0.1 M TPrA. Scan rate was 100 mV/s.

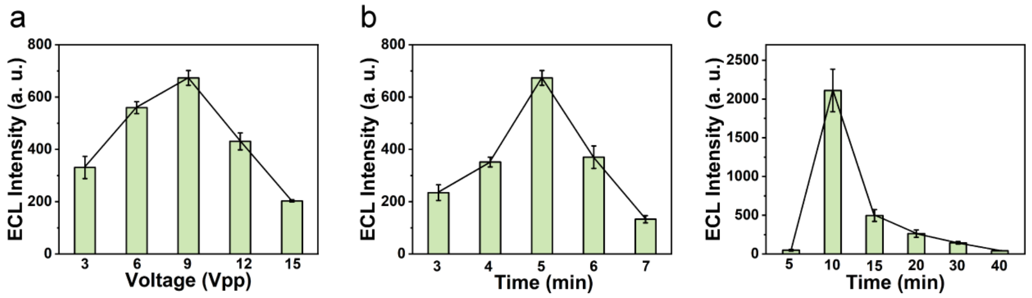

Figure S24 Optimization of experimental conditions. Optimization of V_{pp} of AC (a), AC-driven incubation time of cTnI (b), AC-driven incubation time of miR-499-5p (c).

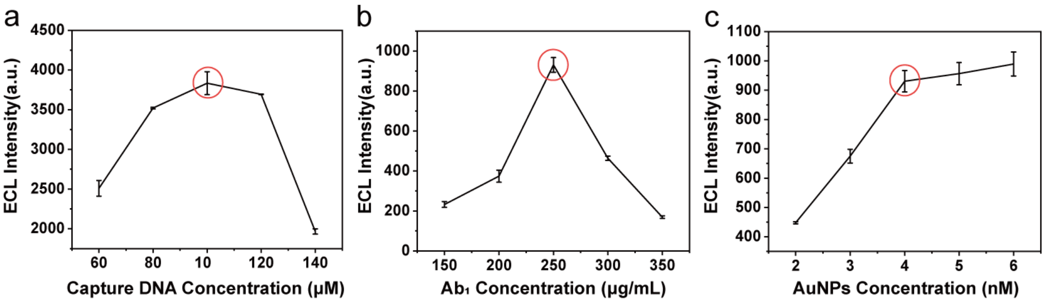

Figure S25 Optimization of experimental conditions. a, b, c, Optimization of concentration of capture DNA (a), Ab_1 (b), AuNPs (c).

Table S7 Sequences of other miRNA interferences.

miRNA	Sequence (5'-3')
miR-208a	UGACGGGCGAGCUUUUGGCCCGGGUUAUACCU GAUGCUCACGUAUAAGACGAGCAAAAAGCUUG UUGGUCA
miR-133	UUGGUCCCCUUCAACCAGCUGU
miR-1	UGGAAUGUAAAGAAGUAUGUA

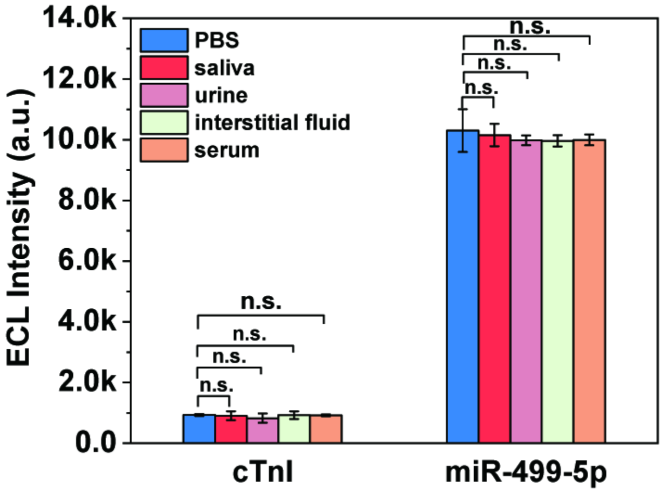

Figure S26 cTnI and miR-499-5p detection in different biological sample solutions (PBS, artificial saliva, urine, interstitial fluid, and serum). Significant difference between ECL signal of PBS and other biological samples was determined by one-way ANOVA followed by a t test. $^*p < 0.05$, $^{**}p < 0.01$, $^{***}p < 0.001$, $^{****}p < 0.0001$ vs. Blank; n.s., no significant.

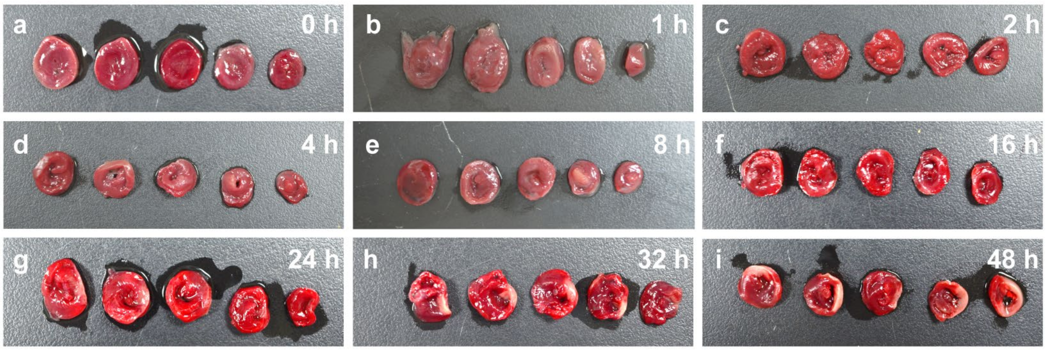

Figure S27 Time-course Infarct photograph of rat models. Once the blood collection was finished, rats were put to death after anesthetized with pentobarbital. Hearts were rapidly removed and sliced transversely into five sections. The hearts were soaked in 10% buffered formalin and frozen in -20 °C for 20 minutes. Five slices were incubated with 1% triphenyl tetrazolium chloride (TTC) at 37°C for 15 minutes under dark conditions to observe the infarction area. The normal heart tissues were stained with red color while infarct heart areas were white.

Table S8 Clinical information on patient samples.

	**AMI**	**VDH**	**CAD**	**Healthy group**
Age				
Median	71	/	/	45
Range	32-95	/	/	27-74
Sex				
Male	13	5	37	20
Female	7	7	19	15

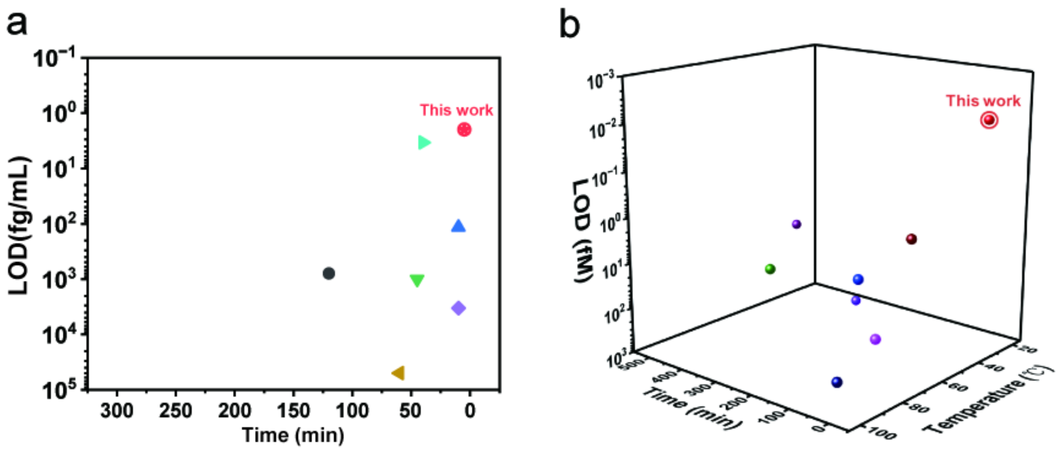

Figure S28 (a) Comparison of ECL-M POCT device (this work) with detection of cTnI with other methods. Incubation time and LOD were estimated and compared with other methods. (b) Comparison of ECL-M POCT device (this work) with detection of miR-499-5p by other methods. Incubation time, reaction temperature, and LOD were estimated and compared with other methods.

Table S9 Comparison of different methods for detection of cTnI in AMI diagnosis.

**Detection method**	**Time (min)**	**Linear dynamic range**	**Limit of detection (LOD)**	**ref**
ECL-M POCT	5	0.01-10 pg/ mL	2 fg/mL	This work
ECL biosensor array	120	1 -10 pg/mL	0.79 pg/mL	[1]
ECL immunoassay	10	0.5–10 pg/mL	0.116 pg/mL	[2]
In_2O_3 FET-based biosensors	45	1-300 pg/mL	1 pg/mL	[3]
graphene-based FET	10	3-60 pg/mL	3.34 pg/mL	[4]
Integrated photothermal-pyroelectric biosensor	60	0.05-100 ng/mL	50 pg/mL	[5]
Dual-signal ECL sensor	40	10 fg/mL- 10μ g/mL	3.39 fg/ mL	[6]

Table S10 Comparison of different methods for amplification-free detection of miRNAs in clinical diagnosis.

Detection method	Target analytes	Time (min)	Temperature (°C)	Limit of detection (LOD)	ref
ECL-M POCT	miR-499	10	25	10 aM	This work
(poly A)-based ECL sensor	miRNA let-7a	65	80	5.1fM	[7]
SCRF biosensor	miRNA let-7a	20	80	70.9 fM	[8]
DNA tetrahedral nanostructure	PC-related miRNA	300	80	10 fM	[9]
3C strategy	miR-499	35	95	0.3 pM	[10]
SNAzyme	miR-133a	300	37	0.3 pM	[11]
SNAzyme	miR-499	480	37	10 fM	[12]
G_4/MOFzymes	miR-499	10	65	1 fM	[13]

References

[1] X. Yang, Y. Zhao, L. Sun, H. Qi, Q. Gao, C. Zhang, *Sensors and Actuators B: Chemical* **2018**, *257*, 60, https://doi.org/https://doi.org/10.1016/j.snb.2017.10.108.

[2] H. Zhao, Q. Lin, L. Huang, Y. Zhai, Y. Liu, Y. Deng, E. Su, N. He, *Nanoscale* **2021**, *13* (5), 3275, https://doi.org/10.1039/D0NR08008J.

[3] Q. Liu, N. Aroonyadet, Y. Song, X. Wang, X. Cao, Y. Liu, S. Cong, F. Wu, M. E. Thompson, C. Zhou, *ACS Nano* **2016**, *10* (11), 10117, https://doi.org/10.1021/acsnano.6b05171.

[4] T. Rodrigues, V. Mishyn, Y. R. Leroux, L. Butruille, E. Woitrain, A. Barras, P. Aspermair, H. Happy, C. Kleber, R. Boukherroub, D. Montaigne, W. Knoll, S. Szunerits, *Nano Today* **2022**, *43*, 101391, https://doi.org/https://doi.org/10.1016/j.nantod.2022.101391.

[5] Z. Yu, H. Gong, Y. Gao, L. Li, F. Xue, Y. Zeng, M. Li, X. Liu, D. Tang, *Small* **2022**, *18* (30), 2202564, https://doi.org/https://doi.org/10.1002/smll.202202564.

[6] C. Hong, P. Zhang, K. Lu, Y. Ji, S. He, D. Liu, N. Jia, *Biosensors and Bioelectronics* **2021**, *194*, 113591, https://doi.org/https://doi.org/10.1016/j.bios.2021.113591.

[7] X. Hua, J. Fan, L. Yang, J. Wang, Y. Wen, L. Su, X. Zhang, *Biosensors and Bioelectronics* **2022**, *198*, 113830, https://doi.org/https://doi.org/10.1016/j.bios.2021.113830.

[8] X. Chen, K. Xu, J. Li, M. Yang, X. Li, Q. Chen, C. Lu, H. Yang, *Biosensors and Bioelectronics* **2020**, *155*, 112104, https://doi.org/https://doi.org/10.1016/j.bios.2020.112104.

[9] D. Zeng, Z. Wang, Z. Meng, P. Wang, L. San, W. Wang, A. Aldalbahi, L. Li, J. Shen, X. Mi, *ACS Applied Materials & Interfaces* **2017**, *9* (28), 24118, https://doi.org/10.1021/acsami.7b05981.

[10] G. Chen, Y. Shen, T. Xu, F. Ban, L. Yin, J. Xiao, Y. Shu, *Biosensors and Bioelectronics* **2016**, *77*, 1020, https://doi.org/https://doi.org/10.1016/j.bios.2015.08.067.

[11] Y. Sun, L. Shi, Q. Wang, L. Mi, T. Li, *Analytical Chemistry* **2019**, *91* (5), 3652, https://doi.org/10.1021/acs.analchem.8b05696.

[12] L. Shi, Y. Sun, L. Mi, T. Li, *ACS Sensors* **2019**, *4* (12), 3219, https://doi.org/10.1021/acssensors.9b01655.

[13] L. Mi, Y. Sun, L. Shi, T. Li, *ACS Applied Materials & Interfaces* **2020**, *12* (7), 7879, https://doi.org/10.1021/acsami.9b18053.
